# Genome-resolved metatranscriptomics unveils distinct microbial functionalities across aggregate sizes in aerobic granular sludge

**DOI:** 10.1016/j.ese.2025.100560

**Published:** 2025-03-25

**Authors:** A.Y.A. Mohamed, Laurence Gill, Alejandro Monleon, Mario Pronk, Mark van Loosdrecht, Pascal E. Saikaly, Muhammad Ali

**Affiliations:** a Department of Civil, Structural & Environmental Engineering, Trinity College Dublin, The University of Dublin, Dublin 2, Ireland; b Department of Biotechnology, Delft University of Technology, Delft, 2629 HZ, the Netherlands; c Department of Chemistry and Bioscience, Center for Microbial Communities, Aalborg University, Denmark; d Environmental Science and Engineering Program, Biological and Environmental Science and Engineering (BESE) Division, King Abdullah University of Science and Technology (KAUST), Thuwal, 23955-6900, Saudi Arabia

**Keywords:** Genome-resolved metatranscriptomics, Aerobic granular sludge, Wastewater treatment, Microbial activity, Differential expression analysis

## Abstract

Microbial aggregates of different sizes in aerobic granular sludge (AGS) systems have been shown to exhibit distinct microbial community compositions. However, studies comparing the microbial activities of different-sized aggregates in AGS systems remain limited. In this study, genome-resolved metatranscriptomics was used to investigate microbial activity patterns within differently sized aggregates in a full-scale AGS plant. Our analysis revealed a weak correlation between the relative abundance of metagenome-assembled genomes (MAGs) and their transcriptomic activity, indicating that microbial abundance does not directly correspond to metabolic activity within the system. Flocculent sludge (FL; <0.2 mm) predominantly featured active nitrifiers and fermentative polyphosphate-accumulating organisms (PAOs) from *Candidatus* Phosphoribacter, while small granules (SG; 0.2–1.0 mm) and large granules (LG; >1.0 mm) hosted more metabolically active PAOs affiliated with *Ca.* Accumulibacter. Differential gene expression analysis further supported these findings, demonstrating significantly higher expression levels of key phosphorus uptake genes associated with *Ca.* Accumulibacter in granular sludge (SG and LG) compared to flocculent sludge. Conversely, *Ca.* Phosphoribacter showed higher expression of these genes in the FL fraction. This study highlights distinct functional roles and metabolic activities of crucial microbial communities depending on aggregate size within AGS systems, offering new insights into optimizing wastewater treatment processes.

## Highlights

• Genome-resolved metatranscriptomics was used to assess microbial activities in aerobic granular sludge (AGS).
• Transcriptomic activity (RNA) showed weak correlation with microbial abundance (DNA).
• *Ca.* Accumulibacter PAOs were more active in granules, whereas *Ca.* Phosphoribacter PAOs were more active in flocs.
• Nitrifying bacteria (AOB and NOB) displayed higher activity levels in flocculent sludge compared to granular sludge.

## Graphical abstract

**Figure:**
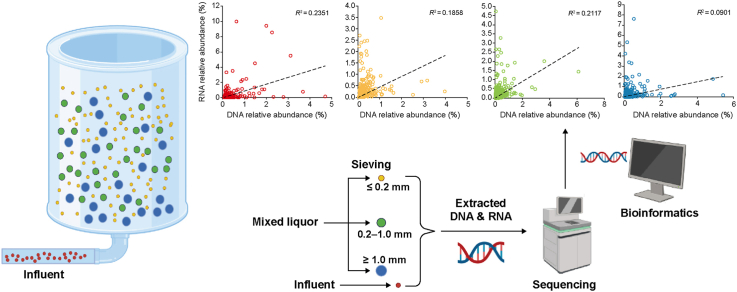

## 1 Introduction

Since its introduction in 1914, conventional activated sludge (CAS) has been crucial for treating domestic and industrial wastewater [1]. The CAS systems require large areas and intensive aeration and have recurrent operational issues such as sludge bulking and foaming [2]. One innovative approach that has gained significant attention in recent years is the implementation of aerobic granular sludge (AGS) systems. The AGS system has exhibited exceptional promise in treating wastewater, offering enhanced nutrient removal and reduced energy consumption and footprint compared to CAS systems [3]. Specifically, the AGS system can reduce the required space by as much as 75 % and energy demand by up to 40 % [4]. Currently, more than 120 full-scale AGS plants are in operation or under construction worldwide [5]. These characteristics highlight the potential for the AGS system to replace CAS systems and become the standard technology for biological wastewater treatment.

In the AGS system, different-sized microbial aggregates (flocs and granules) grow in the same reactor and collectively contribute to the degradation of organic matter and the removal of nutrients from wastewater. Granules are intentionally enriched by selective feeding and retained by selectively wasting flocculent sludge [6,7]. The cyclic feast (anaerobic phase) and famine (aerobic phase) regime, and the high food-to-microorganism (F:M) feeding ratio minimizes diffusion limitations in granules and promotes the growth of slow-growing polyphosphate and glycogen accumulating organisms (PAOs and GAOs). This strategy forms a compact, smooth granular structure [8,9]. The anaerobic feeding phase occurs in an upward plug-flow mode with the influent introduced from the bottom of the reactor. The granules predominantly occupy the lower regions of the reactor during feeding phases, while flocs are more prevalent in the upper sections due to their slower settling velocity than granules. The spatial separation of biomass during feeding creates distinct biological niches due to variations in readily biodegradable substrate availability at specific depths. Soluble, readily biodegradable substrates lead to the growth of granules, while particulate substrates mainly lead to the floc fraction of the AGS system [6]. Furthermore, the microbial communities residing in different-sized microbial aggregates are known to be stratified due to varying redox conditions along the depth of the granules. For example, in the outer layer (up to 100–200 μm) of the microbial granules, typically aerobic microbes such as nitrifiers are present due to oxygen availability in that zone [10], while in the inner layer, PAOs and denitrifies are present due to anaerobic or anoxic conditions [11]. These aspects render the AGS system a unique ecosystem marked by a gradient of selection pressure and different redox conditions [12].

Different-sized microbial aggregates have been proven to vary in their microbial community compositions. For example, species responsible for nitrogen and phosphorus removal were found to be more enriched in large microbial aggregate sizes, while faecal and gut microbes were found to be more enriched in small microbial aggregates [13]. Previous studies have mainly employed 16 S ribosomal RNA (16 S rRNA) amplicon sequencing to explore the microbial community compositions of different-sized microbial aggregates [13]. This method, while easier and less expensive, does come with limitations. These limitations arise from variations in the 16 S rRNA gene copy numbers across species, discrepancies in primer specificity, and biases introduced during the polymerase chain reaction (PCR) amplification process [14]. Also, amplicon sequencing cannot be used to indicate microbial activity.

In this context, metagenomics and metatranscriptomics approaches can complement and overcome the weaknesses of the amplicon-based analyses. These advanced omics tools have been utilized previously to study microbial activities in various biological wastewater treatment systems and provided insightful and fundamental knowledge. For example, genome-resolved metatranscriptomics was employed successfully to study the metabolic activities of *Candidatus* Accumulibacter clades for flocculent sludge in an activated sludge system [15]. Activity-based metaproteomics has also been used previously to characterize the microbial community of larger-sized granules (2 mm) in AGS systems [16]. Metagenomics and metatranscriptomics sequencing are useful tools for exploring the phylogenetic diversity and metabolic capabilities/activities of PAOs. These organisms play a critical role in phosphorus removal due to their ability to store phosphorus as polyphosphate (PolyP). Traditionally, in the anaerobic stage, PAOs take up volatile fatty acids (VFAs) present in the influent and store them as poly-β-hydroxyalkanoates (PHA), using glycogen as a reducing agent and obtaining the required energy through the hydrolysis of intracellularly stored PolyP. In the subsequent aerobic stage, PAOs oxidize the PHA, generating energy for glycogen replenishment, growth, and phosphate uptake [17]. However, different PAO species are hypothesized to exhibit key functional differences in VFA uptake rates, nitrogen-cycling capabilities, and phage defence mechanisms [15]. These functional differences can be explored using genome-resolved metatranscriptomics.

Nevertheless, studies comparing the microbial activities of different-sized aggregates in an AGS system are lacking. Therefore, in this study, a genome-resolved metatranscriptomics was utilized to comprehensively assess the activities of microbial communities within different-sized aggregates present in a full-scale AGS system, including large granules (LG; >1 mm), small granules (SG; 0.2–1 mm), and flocs (FL; <0.2 mm). The primary objectives of this study were to (1) investigate and identify active and non-active microbial populations across different-sized aggregates under varying conditions (anaerobic and aerobic) and (2) examine the metabolic activities of PAOs, focusing on enhanced biological phosphorus removal (EBPR)-related genes. The study focuses on the activity of key functional groups responsible for carbon, nitrogen, phosphorus, and sulphur removal. This work enhances understanding of the role of differently-sized aggregates in AGS system function. It also provides a framework for analysing gene expression patterns in closely related PAOs.

## 2 Materials and methods

### 2.1 Sampling of full-scale AGS plant

Grab samples (300 mL) were collected during three consecutive weeks (*n* = 3) from the Ringsend full-scale AGS wastewater treatment plant (WWTP) in Dublin, Ireland (Supplementary Material Fig. S1). The plant can treat a load of about 1.7 million population equivalents. Design and operational data for the AGS plant, including details on plant design and influent, effluent, and sludge characteristics, are presented in Table S1 (Supplementary Material). During the sampling campaign, the AGS plant maintained stable performance regarding chemical oxygen demand (COD) and nitrogen and phosphorus removal [18]. Samples were taken from the influent (after primary settling and equalization tank) and AGS reactor. The AGS mixed liquor samples were collected from a depth of 4 m at the bottom of the AGS reactor. Samples were collected during both anaerobic (feeding) and aerobic phases, 30 min after the start of each phase to ensure optimal microbial activity. All samples were promptly preserved with RNAlater (Sigma-Aldrich, Merck, USA), shipped on ice, and stored at 4 °C for 24 h. The AGS samples were further sieved into three fractions: FL (<0.2 mm), SG (0.2–1 mm) and LG (>1 mm). This classification is based on established criteria widely used in AGS research [4,13]. These categories are known to represent distinct microbial communities and functional roles. The AGS samples comprise 25 % FL, 53 % SG and 22 % LG (Supplementary Material Table S3). Following this, all samples underwent centrifugation at 8000 rpm for 10 min to obtain a pellet, to which RNAlater (Sigma-Aldrich, Merck, USA) was added in a 1:1 vol ratio. The samples were stored at −80 °C until DNA and RNA extraction.

### 2.2 DNA and RNA extraction and library preparation

The RNAlater was removed from the samples before DNA and RNA extraction, and biomass pellets were washed several times with phosphate buffer solution (PBS). Aliquots of triplicate samples (*n* = 3) were pooled into one sample for each type to perform DNA extraction. Total genomic DNA was extracted from 0.3 g of biomass using FastDNA SPIN Kit for Soil (MP Biomedicals, Santa Ana, CA, USA) following the manufacturer's recommendations. The library was constructed with the VAHTS® Universal Plus DNA Library Prep Kit for Illumina according to manufacturer instructions. The library construction steps consist of DNA fragmentation and end preparation & dA-tailing, adapter ligation, cleanup, library amplification and final cleanup step. Adapter ligation was performed by forward Nextera adapter (3′): “AGATCGGAAGAGCACACGTCTGAACTCCAGTCAC”; and reverse Nextera adapter (5′): “AGATCGGAAGAGCGTCGTGTAGGGAAAGAGTGT”.

Total RNA was extracted from triplicate samples (*n* = 3) using the TRizol Reagent Kit (Invitrogen, Thermo Fisher Scientific, Oregon, USA) according to the manufacturer's guidelines. The TruSeq Stranded Total RNA With Illumina Ribo-Zero Plus rRNA Depletion (Illumina, CA, USA) was used to deplete rRNA in total RNA and construct the library of metatranscriptome sequencing according to manufacturer instructions. The steps of library construction consist of depleting rRNA, fragmenting and denaturing RNA, synthesising first strand cDNA, synthesize second strand cDNA, adenylating 3′ends, ligate adapters, cleaning up libraries, amplifying DNA fragments (PCR), performing second cleanup and check libraries.

The DNA and RNA concentrations and quality of the metagenomic and metatranscriptomic libraries were measured and assessed with Nanodrop (Thermo Fisher Scientific, Oregon, USA), Labchip GX (PerkinElmer, Waltham, Massachusetts, USA) and Qubit dsDNA HS Assay Kit and Qubit 4.0 Fluorometer (Invitrogen, Thermo Fisher Scientific, Oregon, USA). Sequencing of the metagenomic and metatranscriptomic libraries was then performed on the Illumina NovaSeq 6000 platform, using paired-end 150 bp (PE150) sequencing, targeting a sequencing depth of 10 Gb per sample.

### 2.3 Metagenomic assembly and annotation

Metagenomic assembly and recovery of genome workflow were conducted in the Conda environment in the Linux command line interface. Raw reads were quality-assessed using FastQC-v0.11.9 [19] and quality-filtered using Cutadapt v4.3 [20]. The quality filtering step included trimming the adapters and barcode sequencings as well as removing low-quality reads by setting up the minimum quality score (Q-score) and length of the reads to 25 and 100 bp, respectively. Filtered forward and reserve reads of all samples were concatenated separately into a single file and then assembled using MEGAHIT v1.2.9 [21] to generate contigs. The assembled contigs were reformatted, and contigs <2500 bp were eliminated using anvi'o v7.1 command anvi-script-reformat-fasta [22]. A contigs database containing open reading frames (ORFs) and Hidden Markov models (HMMs) were created using anvi-gen-contigs-database and anvi-run-hmms commands on the anvi'o v7.1 software package [22]. The filtered forward and reverse reads for each sample were mapped back to the assembled contigs files using the Maximal Exact Match algorithm of Burrows-Wheeler Aligner (BWA-MEM) [23], and converted to the sequence alignment map (SAM) format and subsequently into a sorted and indexed BAM-files using the samtools algorithm [24]. Metagenome-assembled Genomes (MAGs) were generated from assembled scaffolds by unsupervised binning based on sequence composition, differential coverage, and read-pair linkage using the metabat2 v2.15.0 algorithm [25]. Then, the recovered MAGs were manually refined by the command anvi-refine provided in anvi'o v7.1 [22]. Once the binning collection was ready, the anvi-summarize command in anvi'o v7.1 [22] provided a summary that included details about the MAG completion as well as statistics like mean coverage and variability. MAGs with contamination/redundancy < 10 % qualified for downstream analysis. Relative abundance values of metagenomics read mapped to each MAG were obtained from the “bins_percent_recruitment.txt” file, which was also generated from the anvi-summarize command. These relative abundance values have already been normalized against the genome length of each MAG. Genes of MAGs were functionally annotated using Prokka v1.14.6 [26]. Additional annotation was conducted based on the Kyoto Encyclopedia of Genes and Genomes (KEGG: GhostKOALA), which provides accurate and high-level functions and utilities for organisms and ecosystems [27]. Finally, taxonomic classifications were assigned to MAGs based on the Genome Database Taxonomy (GTDB) using the workflow “classify_wf” on the GTDB-Tk v2.2.6 program [28]. Heatmaps of metagenomic-based relative abundance were produced using the R-package of ampvis2 [29] in R software v3.3.1. Biological functions of the genus-level taxa next to the heatmap were assigned according to the MiDAS field guide [30].

### 2.4 Metatranscriptomic processing and mapping

Generated reads were obtained from the NovaSeq 6000 platform and assessed for quality using FastQC-v0.11.9 [19]. Reads trimming and quality filtering were conducted through Cutadapt v4.3 [20]. The rRNA reads were discarded using SortMeRNA 4.3.6 [31] based on the SILVA rRNA gene database [32]. The remaining filtered mRNA reads were mapped against the genes identified by Prokka for each MAG using Burrows-Wheeler Aligner [23]. Alignments with a sequence identity <97 % were eliminated. The count tables were used to generate gene expressions of microorganisms represented by the MAGs recovered from the samples. These reads were normalized against the gene's lengths. The total expression of each MAG was calculated as the sum of normalized transcriptomic reads for all genes encoded by its representative MAG. Then, the relative abundance of MAG's expression was calculated by dividing the reads count of each MAG by the total reads count across MAGs. A heatmap plot of transcriptomic-based relative abundance was produced for further comparative analysis using the R-package of ampvis2 [29]. For differential gene expression (DGE) analysis, the count tables of gene expression were imported to RStudio (R v3.3.1) and processed using the default DESeq2 workflow [33]. Differential expression analysis was also used to compare the overall expression of MAGs across different conditions using DESeq2 workflow by importing the count tables of overall MAG expression [34]. Differential expression analysis results were visualized using different R packages (ggplot2, ComplexHeatmap [35] and EnhancedVolcano [36]). The principal coordinates analysis (PCoA) plot illustrating the overall similarity of metatranscriptomic-based community composition across samples was generated using a Bray-Curtis dissimilarity distance matrix. Analysis of similarity (ANOSIM) was conducted and visualized using the Vegan package in RStudio (R v3.3.1). Hierarchical clustering, employing the average linkage method, was performed to construct dendrograms representing the clustering of samples.

### 2.5 Data availability

Raw metagenomics and metatranscriptomics sequencing data and MAGs were deposited at the National Center for Biotechnology (NCBI) Sequence under accession number PRJNA1054024.

## 3 Results

### 3.1 Transcriptional activity did not always correlate to MAG abundance

Four samples were processed for metagenomic sequencing corresponding to four types of samples: influent and AGS (FL, SG, LG). A total of 141.77 million paired-end (PE) reads (42.53 Gb) were obtained for metagenomics sequencing, with a minimum yield of 10.38 Gb raw data per sample (Supplementary Material Table S4). A total of 21 samples were processed for metatranscriptomics sequencing corresponding to seven types of samples (*n* = 3) as follows: influent, AGS-anaerobic (FL, SG, and LG), and AGS-aerobic (FL, SG, and LG). A total of 744.87 million PE reads (223.46 Gb) were acquired from metatranscriptomics sequencing with a minimum yield of 9.88 Gb raw sequencing data per sample (Supplementary Material Table S5). A total of 285 MAGs were recovered from assembly and binning (Supplementary Material Table S6). Metagenomic sequencing was aimed at recovering draft near-complete MAGs. Therefore, only one sample was sequenced for each category to facilitate the differential genome binning process. However, triplicate samples were analysed for metatranscriptomics to ensure robust statistical representation and reflect variability in transcriptomic expression.

The composition of the microbial community contained important functional groups responsible for carbon, nitrogen, phosphorus, and sulphur removal/conversions (Supplementary Material Fig. S2). These included PAOs (such as the genera *Tetrasphaera* and *Candidatus* Accumulibacter) and GAOs (including the genera *Propionvibrio*, *Ca.* Competibacter, and *Ca.* Contendobacter). *Ca.* Accumulibacter was the most abundant genera in all the samples for the metagenomics and metatranscriptomics reads and was enriched more in LG, followed by SG, and then FL. Nitrogen cycle microbes such as nitrifiers were presented within the genera *Nitrosomonas*. Furthermore, the microbial community also included genera related to sulphate reduction (e.g., *Acidovorax*) and nitrite reduction, such as *Thiothrix*, *Zoogloea*, *Rhodoferax* and *Sulfuritalea*. As a result, the AGS plant consistently maintained stable performance regarding COD, nitrogen, and phosphorus removal [18].

**Fig. 1:**
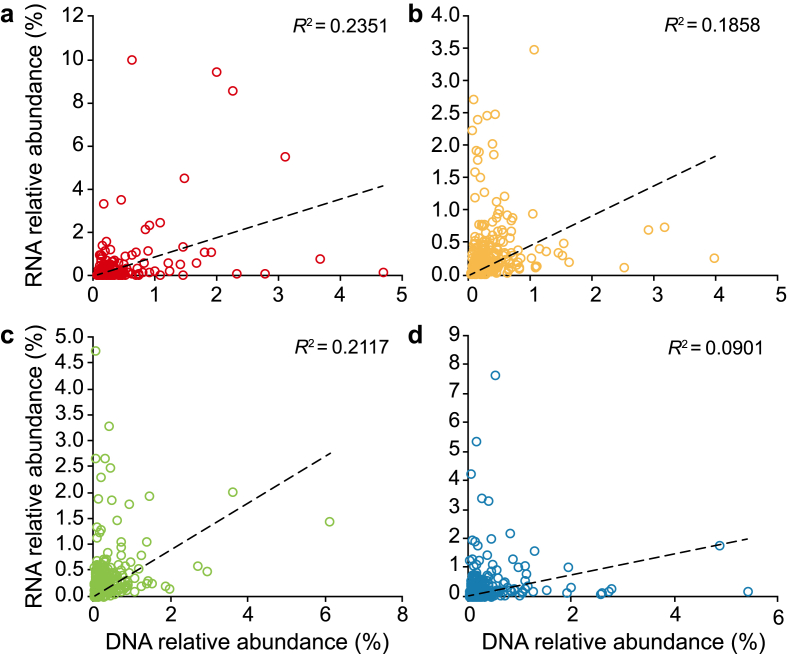
Correlation of relative abundance of RNA and DNA (*n* = 285 species) in influent (**a**), flocs (**b**), small granules (**c**), and large granules (**d**).

Some species showed high MAG abundance but low transcriptomic abundance, while others exhibited the opposite trend (Fig. 1). Therefore, the microbial activity for a certain species is not necessarily proportional to the MAG abundance, and different species can yield different RNA/DNA ratios. Species positioned in the upper left side of each plot have higher activity (RNA/DNA ratios) than the species positioned in the bottom right corner of the plots (Fig. 1). It is important to note that the relative abundances of RNA and DNA were determined using the coverage of mapped reads across the entire genome, rather than relying solely on the coverage of 16 S rRNA genes. This approach ensures that the results are not influenced by variations in 16 S rRNA gene copy numbers.

### 3.2 Important functional groups had different activity levels in different-sized microbial aggregates

**Fig. 2:**
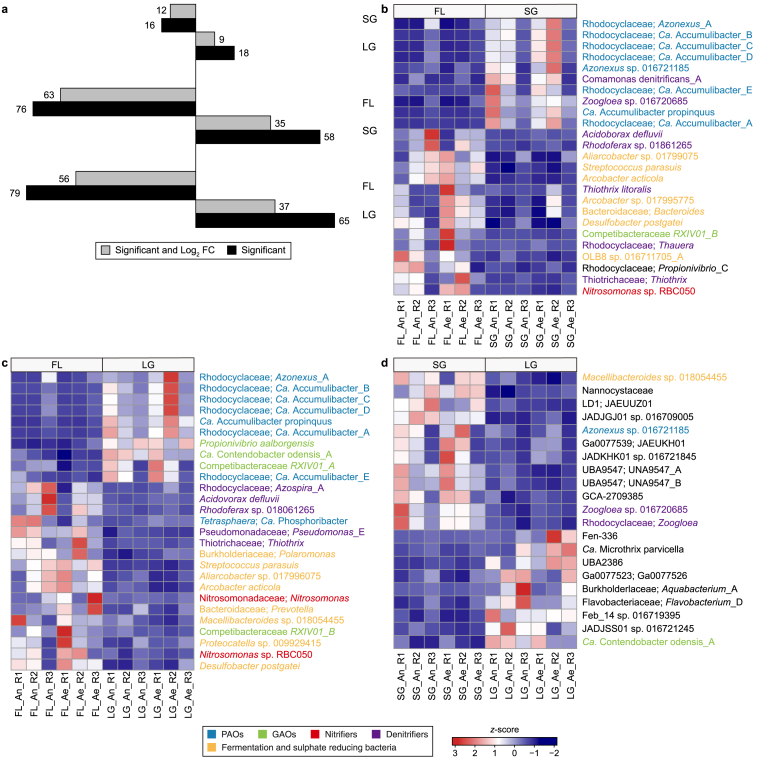
Comparison of microbial activity between different-sized microbial aggregates. **a**, Total number of significant (*P* < 0.05) and differentially expressed/active species (|log_2_ fold change (FC)| ≥ 1 and *P* < 0.05; out of 285 species) between different aggregate sizes (i.e., LG vs. FL, SG vs. FL, LG vs. SG). Numbers of upregulated species for a specific aggregate size are indicated next/aligned to the bars. These numbers can also be interpreted as the number of downregulated species in the compared/opponent aggregate size. For this reason, the bars were flipped/mirrored. **b**, Important differentially expressed species between FL and SG samples. **c**, Important differentially expressed species between FL and LG samples. **d**, all differentially expressed species between SG and LG samples. All listed species pass the threshold of |log_2_ FC| ≥ 1 and *P* < 0.05. Important functional groups among species were coloured based on the group type (i.e., polyphosphate accumulating organisms [PAOs], glycogen accumulating organisms [GAOs], nitrifiers, denitrifies, and fermentation bacteria) according to the MiDAS field guide [30]. FL: flocs; SG: small granules; LG: large granules. An: anaerobic phase; Ae: aerobic phase. Samples were taken in triplicates (R1, R2, R3).

Differential expression analysis using DESeq2 was performed to compare the transcriptional activity (at the genome level) between different-sized microbial aggregates (FL, SG, and LG) (Fig. 2). When comparing SG with FL, there were 58 species significantly (*P* < 0.05) upregulated in SG (35 species with |log_2_ fold change (FC) ≥ 1) versus 76 species significantly upregulated in FL (63 species with |log_2_ FC| ≥ 1) (Fig. 2a). Samples of the SG had more active PAOs (six species: all belonging to the genus *Ca*. Accumulibacter; |log2 FC| ≥ 1, *P* < 0.05) than FL samples (Fig. 2b). Two members of the genus *Azonexus* (formerly *Dechloromonas*), which are considered to be PAOs [[37], [38], [39]], were also found to be more active in SG than FL. In particular, the species *Azonexus* sp. 016721185 (known as *Ca.* Dechloromonas phosphorivorans) was proven to play an important role in EBPR systems, exhibiting a complete EBPR phenotype [37]. Conversely, FL samples contained more active GAOs (two species) and nitrifiers (one species; *Nitrosomonas* sp. RBC050) than SG samples (|log2 FC| ≥ 1, *P* < 0.05). When comparing LG with FL, there were 65 species significantly (*P* < 0.05) upregulated in LG (37 species with |log_2_ FC| ≥ 1) versus 79 species significantly upregulated in FL (56 species with |log_2_ FC| ≥ 1) (Fig. 2a). Similar to SG, six PAO species (all belonging to the genus *Ca*. Accumulibacter) were more active in LG than FL samples (one species; *Ca*. Phosphoribacter Previously named as *Tetrasphaera*) (Fig. 2c), indicating that *Ca*. Accumulibacter prefers to grow in LG, while *Ca*. Phosphoribacter had a preference for growing in FL. In addition, LG contained one putative active PAO belonging to the genus *Azonexus.* LG samples also contained more active GAOs (three species; |log2 FC| ≥ 1*, P* < 0.05) than the FL samples (one species). Interestingly, all nitrifiers were more active (|log2 FC| ≥ 1, *P* < 0.05) in FL samples (two species of the genus *Nitrosomonas;* Fig. 2c). In general, FL samples contained many active denitrifying bacteria as compared to SG and LG (|log2 FC| ≥ 1, *P* < 0.05), including members of the genera *Rhodoferax*, *Acidovorax*, *Thiothrix*, *Pseudomonas*, *Thauera* and *Azospira* [30]. Some members of the genera *Thiothrix* and *Thauera* have been indicated as PAOs [40,41]. *Ca*. Accumulibacter bacteria found to be active in the SG and LG samples can also be argued to perform denitrification [39]. Compared to SG and LG, FL samples also contained many species with higher activity, like those found more active and abundant in the influent wastewater, including fermentation bacteria (i.e., species of the genera *Macellibacteroides*, *Paludibacter*, *Prevotella*, *Proteocatella*, *Streptococcus*, *Arcobacter*, *Aliarcobacter*, *Cloacibacterium*, *OLB8,* and *Bacteroides*) and sulphate-reducing organisms (*Desulfobacter* and *Acidovorax*). Influent samples were highly enriched with fermentative and sulphate-reducing genera such as *Arcobacter* (15.1 %), *Macellibacteroides* (14.9 %), *Prevotella* (11.0 %), *Bacteroides* (6.8 %), *Paludibacter* (3.2 %), *Acidovorax* (2.4 %) (Supplementary Material Fig. S2). This suggests a high similarity between the influent and FL samples, which is supported by the PCoA plot (Supplementary Material Fig. S3a). There were not many differences in microbial activity between the SG and LG samples compared to their differences to the FL samples (Fig. 2d), indicating high similarity between SG and LG on a transcriptomic level, which is also supported by PCoA plot (Supplementary Material Fig. S3a) and analysis of similarity (Supplementary Material Fig. S3b). There were only 18 species significantly (*P* < 0.05) upregulated in the LG (nine species with |log_2_ FC| ≥ 1) versus 16 species significantly upregulated in the SG (12 species with |log_2_ FC| ≥ 1) (Fig. 2a). None of these species were related to the important functional group except one GAO species (*Ca*. Contendobacter odensis_A) which was more active in LG than SG, and two members of the genus *Zoogloea* (denitrifying bacteria) which were more active in the SG than LG (Fig. 2d).

There were no significant differences in transcriptional activity between anaerobic and aerobic conditions of different-sized microbial aggregates (FL, SG, LG, and global) at the genome level (total gene expression of a particular genome) (Supplementary Material Fig. S4a). However, there were significant differences between anaerobic and aerobic conditions at the individual gene level, suggesting distinct metabolic pathways/metabolism during anaerobic and aerobic conditions (Supplementary Material Fig. S4b). In general, more genes were significantly expressed aerobically than anaerobically (Supplementary Material Fig. S4b). Although DESeq2 analysis requires a minimum of duplicate experimental data, our study was conducted using biological triplicates (*n* = 3) to enhance the robustness of the dataset. While this approach provides a robust snapshot of microbial dynamics under normal conditions, a more extensive sampling design to capture seasonal or longer-term variability would offer further insights. This study can serve as a benchmark for future research, and we recommend incorporating a higher number of biological replicates, along with database-supported validations, to further mitigate the impact of this limitation. Furthermore, a more detailed integration of transcriptomics with macroscopic processes involved in C, N, and P metabolism and energy cycling modes represents an important avenue for future research.

Total PAOs had higher transcriptional activity (relative expression) in the SG and LG than in the FL and influent samples (Supplementary Material Fig. S5a). Conversely, total nitrifiers (including ammonium-oxidizing bacteria [AOB] and nitrite-oxidizing bacteria [NOB]) had higher transcriptional activity in the influent and FL than in the SG and LG samples (Supplementary Material Fig. S5a). Total GAOs also had higher transcriptional activity in the influent than FL, SG, and LG, but there were not many differences between the different-sized microbial aggregates (Supplementary Material Fig. S5b). Interestingly, transcriptional activity was not always proportionally correlated to MAG abundance. For example, total nitrifiers had low MAG abundance but higher transcriptional activity in the influent, as opposed to the SG, which had higher MAG abundance but low transcriptional activity (Supplementary Material Fig. S5a). Similarly, total GAOs had lower MAG abundance but higher transcriptional activity in the influent than LG, which had higher MAG abundance but relatively lower transcriptional activity (Supplementary Material Fig. S5b).

**Fig. 3:**
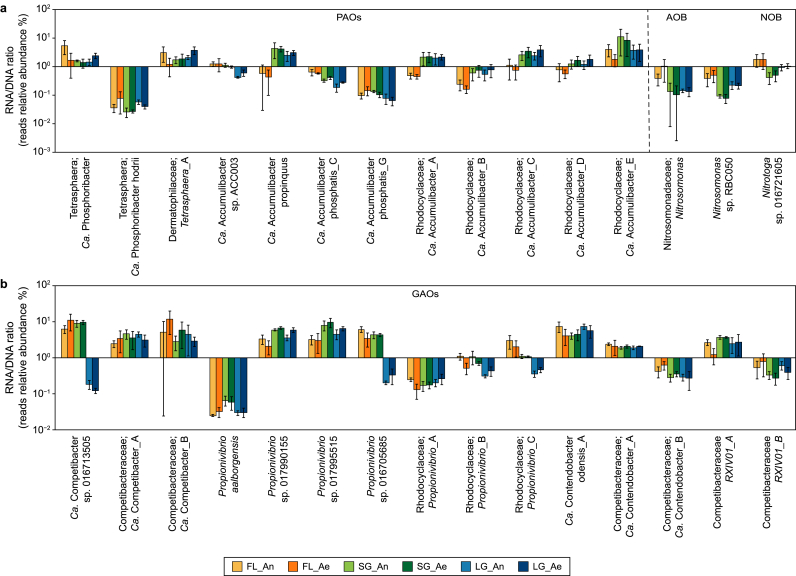
Transcriptomic (RNA) relative expression to MAG (DNA) relative abundance ratios of the species of important functional groups in flocs, small granules, and large granules during anaerobic (An) and aerobic (Ae) phases. **a**, Polyphosphate accumulating organisms (PAOs) and nitrifiers (ammonium-oxidizing bacteria [AOB] and nitrite-oxidizing bacteria [NOB]); **b**, Glycogen accumulating organisms (GAOs).

It is pertinent to mention that our DESeq2 analysis (Fig. 2) was conducted using transcriptomic data without normalization against metagenomic data. One might argue that conventional PAOs have higher activity in granular sludge because they have higher MAGs relative abundance. For a specific species, the absolute abundance of RNA signifies its overall activity, while the absolute abundance of MAG (DNA) indicates its population size. Consequently, the RNA/DNA ratio serves as a normalized indicator of the species activity per unit. When relative abundance is used instead of absolute abundance to calculate the ratio, it is critical to judge whether a certain species is active or inactive. Instead, we can compare the relative activity of one species to other species or the relative activity of one species under different conditions. Using this approach (RNA/DNA), we also found that the conventional PAOs *Ca.* Accumulibacter had higher activity (RNA/DNA) in SG and LG than in FL samples in most of the cases (Fig. 3a). The FL samples contained more active (RNA/DNA) fermentative PAOs such as *Ca.* Phosphoribacter and *Tetrasphaera.* Interestingly, GAOs which had higher MAG abundance in LG samples had lower (RNA/DNA) ratios in LG than FL and SG in many cases (Fig. 3b). Nitrifiers, including AOB and NOB, had higher RNA/DNA ratios in FL than in SG and LG which agreeing to DESeq2 findings.

Within each functional group, some individual species had high relative expression (activity) to MAG abundance ratios (RNA/DNA), and some had low ratios (Fig. 3), indicating that the relative activity can be different significantly between the species of the same genera and functional group. For example, species of *Ca*. Accumulibacter propinquus, *Ca.* Phosphoribacter and *Ca.* Contendobacter odensis_A had a high relative expression to MAG abundance ratios, while species of *Ca.* Accumulibacter phosphatis_G, *Ca.* Phosphoribacter hodrii and *Propionvibrio aalborgensis* had low relative expression to MAG abundance ratios (Fig. 3).

### 3.3 Metabolic activities of PAOs in different-sized microbial aggregates

**Fig. 4:**
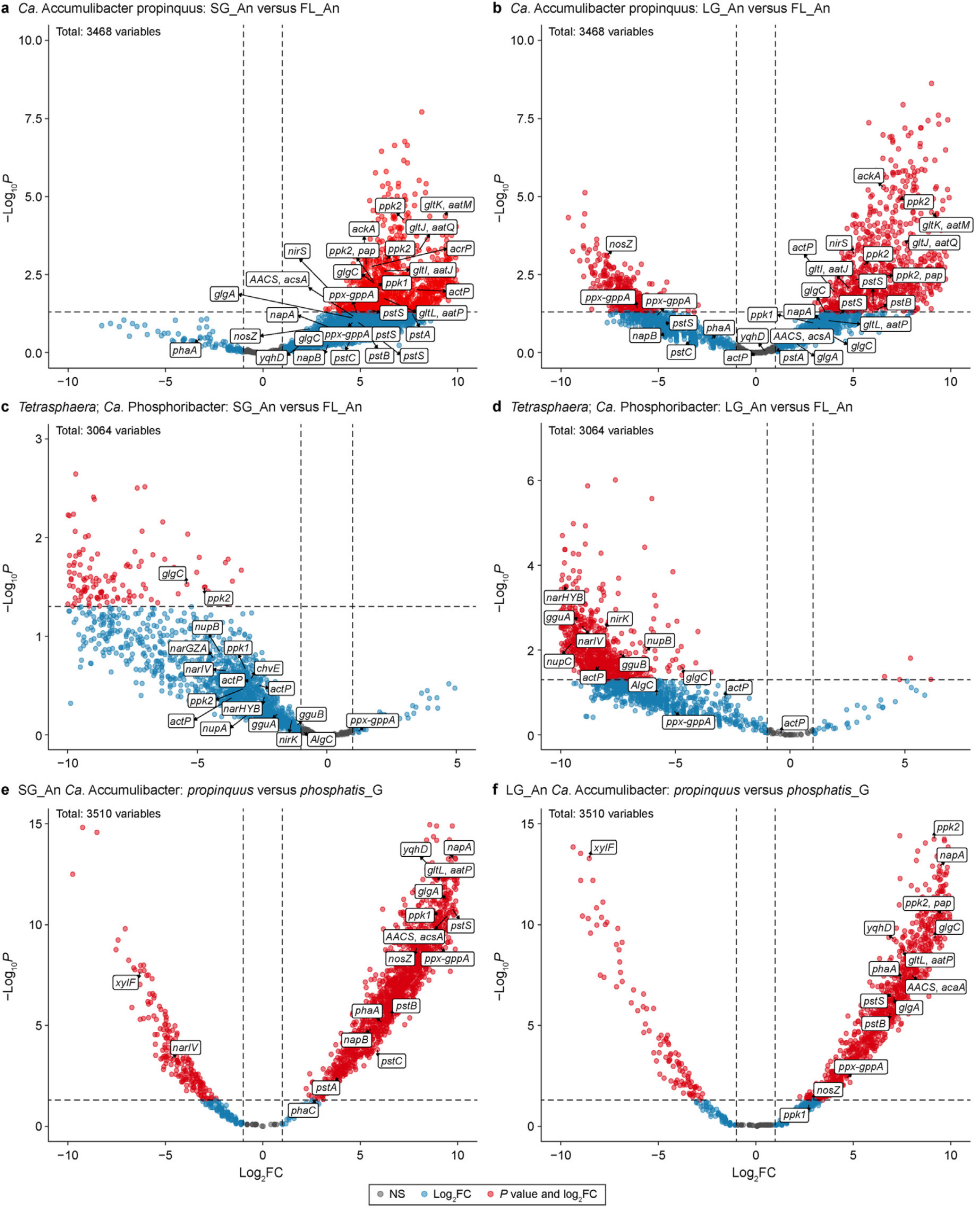
Comparison of metabolic/transcriptional activities of different polyphosphate accumulating organisms in different-sized microbial aggregates using differential genes expression analysis (DESeq2). **a**–**b**, Comparisons of gene expression of *Ca*. Accumulibacter propinquus species between SG and FL (**a**) and between LG and FL (**b**) in the anaerobic/anoxic feeding phase. **c**–**d**, Comparison of gene expression of *Ca.* Phosphoribacter species between SG and FL (**c**) and between LG and FL (**d**) in the anaerobic/anoxic feeding phase. Genes with negative log_2_ fold change (FC) indicate they were downregulated in the SG or LG (or upregulated in the FL). Positive log_2_ FC values suggest that these genes were upregulated in either the SG or LG. **e**–**f**, Comparison of gene expression between *Ca.* Accumulibacter propinquus and *Ca.* Accumulibacter phosphatis_G in SG (**e**) and LG (**f**) in the anaerobic/anoxic feeding phase. Genes with negative log_2_ FC indicate they were upregulated in *Ca.* Accumulibacter phosphatis_G. While positive log_2_ FC values indicate they were upregulated in *Ca.* Accumulibacter propinquus. Genes were coloured based on the level of significance and degrees of variation, either as |log_2_ FC| ≥ 1 or meeting both |log_2_ FC| ≥ 1 and *P* < 0.05 or meeting none of the previous conditions as non-significant (NS). Only important genes related to phosphate-related metabolism were labelled in the plots. FL: flocs; SG: small granules; LG: large granules. An: anaerobic phase; Ae: aerobic phase. Horizonal and vertical dotted lines representing cut-off values of *P* = 0.05 and |log_2_ FC| = 1, respectively.

Twelve PAO species were detected in the current study, nine belonging to the genus *Ca*. Accumulibacter and three belonging to *Tetrasphaera* and *Ca.* Phosphoribacter. The metabolic activities of these PAOs were examined by carrying out direct comparisons at the gene level between the different-sized microbial aggregates. *Ca*. Accumulibacter propinquus (genome completeness 96 % and redundancy 7 %) was the most active PAO in the current study (Supplementary Material Fig. S5a). By looking at the anaerobic metabolism of this species, many more genes were significantly expressed in the LG and SG compared to the FL samples, indicating that *Ca*. Accumulibacter propinquus was more active in granular sludge as compared to flocculant sludge (Fig. 4a and b). These genes include important genes related to biological phosphorus removal such as polyphosphate kinase genes (*ppk1, ppk2, ppk2-pap*) and high-affinity phosphate transporters (*pstSCAB*) [39]. Interestingly, *Ca.* Phosphoribacter species (genome completeness 94.4 % and redundancy 1.4 %) had many biological phosphorus uptake-related genes, which were more significantly expressed in flocculant sludge during the anaerobic/anoxic feeding phase as compared to granular sludge (Fig. 4c and d), especially genes involved in denitrification in wastewater such as nitrate reductase (*narGHI*) and nitrite reductase (*nir*). This indicates that *Ca.* Phosphoribacter was more active anaerobically/anoxically in the FL than in the SG and LG samples as opposed to *Ca*. Accumulibacter propinquus. These organisms play a crucial role in biological phosphorus uptake and denitrification, interconnected processes under specific conditions. The significant expression of denitrification genes highlights the metabolic versatility of *Ca.* Phosphoribacter enables it to simultaneously contribute to phosphorus removal and nitrogen reduction, emphasizing the dual role of *Ca.* Phosphoribacter species as denitrifying phosphorus-accumulating organisms (DPAOs) [39,42]. Interestingly, *Ca.* Accumulibacter phosphatis_G, which had the highest metagenomics-based abundance among the PAOs (Supplementary Material Fig. S5a), had much less activity than *Ca*. Accumulibacter propinquus. This is clear from Fig. 4e and f, which indicates that *Ca*. Accumulibacter propinquus had more PAO-related genes significantly expressed in the SG and LG than *Ca.* Accumulibacter phosphatis_G. These genes also include PHA accumulation genes (*phaABCZ*), polyphosphate kinase genes (*ppk1, ppk2, ppk2-pap*), and high-affinity phosphate transporters (*pstSCAB*). This indicates that different PAO species had different activity, such as different accumulation/degradation rates of PHA/polyphosphate.

## 4 Discussion

### 4.1 Discrepancy between transcriptional activity and MAG abundance

The current study shows that the microbial community composition of the AGS system differs between metagenomics and metatranscriptomics-based analysis techniques (Supplementary Material Fig. S2; Fig. 1). The results show that the correlation of transcriptomics (RNA) relative abundance with MAGs (DNA) relative abundance in influent and AGS system was not strong (*R*^2^ < 0.25), especially for large microbial aggregates (Fig. 1). These discrepancies may arise from transcriptional regulation differences between microbial species, which often result in variations in transcript abundance that are independent of MAG abundance. The effects of transcription factors, enhancers, silencers, and other regulatory elements, gene copy number variation, and post-transcriptional modification could induce the reduction of the correlation between the genome and the transcriptome [43,44]. In addition, Environmental factors, such as substrate availability (e.g., access to slowly vs. readily degradable substrate), oxygen levels, and nutrient gradients, significantly influence gene expression levels and may cause differences in activities, creating unique metabolic niches within microbial aggregates [45]. Furthermore, organisms exhibiting high transcriptional plasticity may display disproportionately elevated transcript levels compared to their genomic representation, reflecting differential activities across functional microbial groups [46]. Prior research has also demonstrated that various sequencing and meta-omics techniques can offer distinct perspectives on microbial community composition. For instance, Kleikamp et al. [16] conducted a study on the microbial composition of granular sludge from three different WWTPs utilising 16 S rRNA, metagenomics, and metaproteomics sequencing. Their investigation revealed notable differences in the relative abundances of bacteria across the different methods, particularly at lower taxonomic levels. Mei et al. [47] and Matar et al. [48] adopted the method of using the ratio of 16 S rRNA and 16 S rRNA gene (16 S rDNA) (rRNA/rDNA) for individual species to identify active species in a full-scale anaerobic digester and lab-scale membrane bioreactor, respectively. Similar to our study, the rRNA/rDNA approach yielded different ratios for different species across samples.

When using relative expression or abundance instead of absolute values to calculate ratios, it becomes crucial to determine whether a species is genuinely active or inactive, as DNA and RNA yields can differ across samples. Alternatively, we can assess a species' relative activity by comparing it to other species. Moreover, there is no universal RNA/DNA threshold to definitively distinguish active from inactive species, as this threshold can vary between species. This variability highlights the importance of context-specific assessments when interpreting RNA/DNA ratios.

### 4.2 Distinct microbial communities and activities in flocculent and granular sludge

Our differential expression analysis revealed that SG and LG samples exhibited significant similarity in metatranscriptomics-based community composition compared to FL samples (Fig. 2). In contrast, the FL biomass showed greater resemblance to influent communities, as it contained numerous active fermentative and sulphate-reducing bacteria that were abundantly active in the influent. These bacteria probably grew in the sewage network as a biofilm, then detached and thrived in the influent by slowly breaking down biodegradable substances, converting them into readily biodegradable substrates essential for PAOs and GAOs in the AGS system. The high similarity between influent and FL samples likely occurred because the influent particulates, including bacteria, are easily incorporated in the flocculent sludge fraction instead of granular sludge. Furthermore, the granules mainly grow on soluble and easy hydrolysable substrates [6], i.e., in granules, most of the biomass is actively growing biomass and hardly consists of material from the influent. Nevertheless, the porosity of the granular matrix would allow the immigration of micron-sized particles like microbes from the influent [49]. The high similarity between influent and flocculent communities is also possibly since flocculent sludge (FL) has shorter SRTs than granular sludge (SG and LG). When the SBR process removes excess sludge, smaller and less dense flocculant microbial aggregates are selectively taken out, while the larger and more dense granular microbial aggregates are retained within the reactor, thereby having longer SRTs. Vuono et al. [50] observed that microbial communities in a full-scale CAS WWTP were more affected by immigration when operated with short SRTs than long SRTs. These results indicate that the granular sludge (SG and LG) community did not merely reflect the flocculent sludge (FL) community. Instead, each community exhibited distinct microbial compositions and activities, emphasizing the unique ecological niches within the AGS system. Also, these findings indicate that flocculent sludge primarily consists of growing microbial aggregates rather than broken granular particles. These findings align with the previous study by Ali et al. [13], which also observed differences in microbial community compositions between granular and flocculent sludges.

Owing to differences in settling velocity between FL, SG, and LG, segregation occurs in the sludge bed post-settling phase, with SG and LG positioned at the bottom, followed by FL at the top. The influent introduced anaerobically in a plug flow regime from the bottom of the reactor exposes the SG and LG to readily biodegradable substrates. A combination of highly biodegradable substrate availability and anaerobic feeding leads to the selection of PAOs in the granular sludge [13]. We observed that PAOs became progressively enriched, more abundant, and more active with increasing microbial aggregate size (Fig. 2; Supplementary Material Fig. S5a). Similar observations have been documented where larger microbial aggregates hosted important functional organisms [12,51].

### 4.3 Activity levels of important functional groups vary across different-sized microbial aggregates

Our study unveiled a unique finding: *Ca.* Phosphoribacter exhibited higher activity in FL than SG and LG. Placing flocculant sludge above the sludge bed creates an environment conducive to the fermentative PAO *Ca.* Phosphoribacter. This is influenced by the presence of slowly biodegradable substrates, as readily biodegradable substrates such as VFAs have already been consumed by *Ca.* Accumulibacter in granular sludge located at the bottom of the sludge bed [13,[52], [53], [54]]. The flocculent sludge layer slowly contains hydrolysable material from the influent, which is hydrolysed and converted to amino acids and sugars. *Ca.* Phosphoribacter and *Tetrasphaera* are frequently found in WWTPs, treating slowly biodegradable and complex wastewater [55]. Their metabolic flexibility encompasses a broader range of substrates, such as amino acids and sugars (hydrolysis products). *Ca.* Phosphoribacter and *Tetrasphaera* are able to uptake and utilize these products through fermentative processes, in contrast to conventional PAOs like *Ca.* Accumulibacter, which does not have fermentation capabilities [56]. Therefore, *Ca.* Phosphoribacter and *Tetrasphaera* are more present in niches where hydrolysis and fermentation are the rate-limiting conversions (top of the reactor; FL), whereas *Ca.* Accumulibacter is located in places with enough VFA (bottom of the reactor; SG and LG). These findings were supported by gene differential expression analysis, which showed that *Ca.* Accumulibacter had many biological phosphorus uptake-related genes that were more significantly expressed in granular sludge (SG and LG) than in flocculent sludge (FL), whereas *Ca.* Phosphoribacter demonstrated the opposite. These included PHA accumulation genes (*phaABCZ*), polyphosphate kinase genes (*ppk1*, *ppk2*, *ppk2-pap*) and high-affinity phosphate transporters (*pstSCAB*). Future research should validate these findings through basic batch experiments to determine whether the differences in polyphosphate and PHA contents in different-sized microbial aggregates of AGS align with the expression trends of polyphosphate kinase genes and PHA accumulation genes.

PAOs, such as *Ca.* Accumulibacter, are favoured in granular sludge (SG and LG, due to their ability to uptake and store VFAs as PHA and subsequently release phosphate during anaerobic phases. Conversely, GAOs, such as *Ca.* Competibacter, can outcompete PAOs under conditions where the influent wastewater has a high COD/P ratio, as they utilize VFAs without contributing to phosphorus removal. These GAOs had higher relative MAG abundance in LG than in FL and SG. However, our results highlighted higher activity among specific GAOs in the influent, such as the fermentative GAOs *Propionvibrio* (*Propionivibrio* sp. 1799155 and *Propionivibrio* sp. 17995515), along with Competibacteraceae *RXIV01_B* (Supplementary Material Fig. S5b). The higher COD/P ratio (i.e., >100 mg COD per mg P) in the raw wastewater likely facilitated GAO proliferation in the sewer network [55]. Their presence in the influent is likely due to their detachment from the biofilm grown in the sewer pipes. These GAOs are considered potential competitive or cooperative organisms. The presence of fermentation products, such as VFAs, can play a crucial role in driving microbial interactions. Depending on the availability of carbon sources and other environmental factors, VFAs can drive either competition or cooperation between PAOs and GAOs. Competition often arises under limited carbon availability, while synergistic interactions may occur in systems with sufficient VFAs [57]. The high abundance of fermentation bacteria in both the influent and FL suggests that significant amounts of organic matter underwent fermentation, producing sufficient VFAs in the sewers and the AGS system. Specific fermentation products such as acetate and propionate can enable GAOs and PAOs to collectively contribute to metabolic processes like phosphorus uptake and storage. Rubio-Rincón et al. [58] reported a synergistic relationship in an EBPR system where GAOs reduced nitrate to nitrite, which PAO I utilized for anoxic phosphate uptake.

Our results also exhibited a greater relative activity of nitrifiers (AOB and NOB) in influent and FL as opposed to SG and LG (Fig. 2). However, they were more enriched (as MAGs abundance) in SG samples (Supplementary Material Fig. S5a). The presence of AOB and NOB in the influent suggests the existence of temporarily aerated niches in the biofilm on the gravity sewer, potentially caused by variable water depth. Bacteria detaching from the biofilm in the sewer pipes might enter the sewage and influent. Additionally, these nitrifiers could originate from sludge thickeners with high suspended solids in the return water to the treatment plant, especially since the influent samples were collected after the equalization tank, just before the AGS reactor. Aerobic nitrifiers demonstrate higher relative activity within smaller-sized microbial aggregates, attributed to their larger surface area to volume ratio, resulting in increased oxygen diffusion relative to biomass amount into the small aggregates or flocs [59]. Similarly, a higher relative abundance of nitrifiers was detected in smaller-sized microbial aggregates in a granular-based anammox bioprocess [60,61]. Furthermore, despite efficient nitrogen removal in the studied AGS plant, nitrifiers were observed to be less abundant (∼1 %) compared to other vital functional groups. This is because autotrophic nitrifiers have a very low biomass yield per unit of oxygen consumed compared to heterotrophs. Earlier studies also highlighted the probability of low nitrifier abundance but with potentially high specific activity within these engineered ecosystems [13,62]. It is plausible that these systems might have undiscovered or unidentified nitrifiers [63]. Interestingly, FL samples contained many active denitrifying bacteria compared to SG and LG, including members of the genera *Rhodoferax*, *Acidovorax*, *Thiothrix*, *Pseudomonas*, *Thauera* and *Azospira*. While flocs generally have better oxygen transfer, localized microenvironments within the flocs can create anaerobic or low-oxygen nitrite/nitrate-rich zones conducive to the growth of denitrifying bacteria [64,65]. These conditions are influenced by substrate availability, microbial activity, and oxygen diffusion [64,65]. Nevertheless, these genera exhibited lower abundances compared to *Ca.* Accumulibacter and *Ca.* Competibacter, which were highly abundant in SG and LG, possess the denitrification capacity [39].

### 4.4 Study implications and application prospects

This study offers valuable insights for engineers involved in designing and operating AGS systems, guiding them in selecting optimal microbial aggregate sizes to enhance the functional performance of the system. For example, large granules are preferable for WWTPs treating high phosphorus concentrations and receiving waste streams enriched with volatile fatty acids (e.g., potato and dairy industries). In contrast, flocs can remove phosphorus in the presence of slowly biodegradable organic matter and complex substrates like sugars and amino acids. Additionally, flocs can be beneficial for treating wastewater enriched with ammonium through nitrification, while granular sludge can provide anoxic conditions for denitrification. Selection and rejection pressures can be applied to control aggregate sizes. By strategically removing waste sludge from appropriate heights within the sludge bed, undesired aggregate sizes can be rejected while desired sizes are retained. This operational technique takes advantage of the natural stratification due to differences in settling velocities among aggregates of various sizes.

## 5 Conclusions

This study showed that metagenomics and metatranscriptomics-based analysis provided different perspectives on the microbial ecology of the AGS system.

• The relationship between the transcriptomic (RNA) relative abundance and MAG (DNA) relative abundance was not correlated, implying that the microbial activity for a certain species is not necessarily relative to the MAG abundance. For example, some species may have high RNA/DNA ratios, and some may have low RNA/DNA ratios.
• Differential expression analysis revealed that SG and LG exhibited considerable similarity in their microbial activity, while both differed significantly from FL samples.
• Granular sludge (LG and SG) contained active conventional PAOs (*Ca.* Accumulibacter), whereas flocculent sludge contained active nitrifiers and fermentative PAOs (*Ca.* Phosphoribacter and *Tetrasphaera*).
• The study also revealed that influent wastewater contained more active fermentative and sulphate-reducing organisms and some nitrifiers and GAOs, such as some species of the genus *Propionvibrio.*
• This study also showed that different PAO species have different metabolic activities within the same microbial aggregate size. For example, *Ca.* Accumulibacter propinquus had more PAO-related genes significantly expressed in SG and LG than *Ca.* Accumulibacter phosphatis_G, although the latter species had higher MAG abundance.

## CRediT authorship contribution statement

**A.Y.A. Mohamed:** Writing – review & editing, Writing – original draft, Software, Methodology, Investigation, Funding acquisition, Formal analysis, Data curation, Conceptualization. **Laurence Gill:** Writing – review & editing, Writing – original draft, Resources, Funding acquisition. **Alejandro Monleon:** Writing – review & editing, Investigation, Conceptualization. **Mario Pronk:** Writing – review & editing. **Mark van Loosdrecht:** Writing – review & editing, Writing – original draft. **Pascal E. Saikaly:** Writing – review & editing, Writing – original draft, Funding acquisition, Conceptualization. **Muhammad Ali:** Writing – review & editing, Writing – original draft, Supervision, Project administration, Methodology, Funding acquisition, Formal analysis, Data curation, Conceptualization.

## Declaration of competing interest

The authors declare the following financial interests/personal relationships which may be considered as potential competing interests: Muhammad Ali reports financial support was provided by 10.13039/501100002081Irish Research Council. A.Y.A. Mohamed and Laurence Gill reports financial support was provided by 10.13039/501100002081Irish Research Council. Muhammad Ali reports administrative support was provided by Irish Water. Muhammad Ali reports administrative support was provided by Celtic Anglian Water. Muhammad Ali reports administrative support was provided by Haskoning DHV Nederland BV. If there are other authors, they declare that they have no known competing financial interests or personal relationships that could have appeared to influence the work reported in this paper.
