# Supplementary material for: Genome-resolved metatranscriptomics unveils distinct microbial functionalities across aggregate sizes in aerobic granular sludge
Source: Environ Sci Ecotechnol. 2025 Mar 25;25:100560. doi: 10.1016/j.ese.2025.100560 (PMC11999188; doi:10.1016/j.ese.2025.100560)
Supplement: Multimedia component 1 [file mmc1.docx]

**Genome-Resolved Metatranscriptomics Unveils Distinct Microbial Functionalities Across Aggregate Sizes in Aerobic Granular Sludge**

**A.Y.A. Mohamed ^a^, Laurence Gill ^a^, Alejandro Monleon ^a^, Mario Pronk ^b^, Mark van Loosdrecht ^b,c^, Pascal E. Saikaly ^d^, Muhammad Ali ^a,*^**

^a^ Department of Civil, Structural & Environmental Engineering, Trinity College Dublin, The University of Dublin, Dublin 2, Ireland

^b^ Department of Biotechnology, Delft University of Technology, Delft 2629 HZ, The Netherlands

^c^ Department of Chemistry and Bioscience, Center for Microbial Communities, Aalborg University, Denmark

^d^ Environmental Science and Engineering Program, Biological and Environmental Science and Engineering (BESE) Division, King Abdullah University of Science and Technology (KAUST), Thuwal 23955-6900, Saudi Arabia

*Correspondence: M Ali, Department of Civil, Structural & Environmental Engineering, Trinity College Dublin, The University of Dublin, Dublin 2, Ireland. E-mail: [Muhammad.ali@tcd.ie](mailto:Muhammad.ali@tcd.ie)

**Table S1** Design and operational parameters for Ringsend Wastewater Treatment Plant.

| **Parameter** | **Value** | **Unit** |
| --- | --- | --- |
| **Flows and capacity** | | |
| Population Equivalent | 1.7 million | PE |
| Organic Capacity - Design / As Constructed | 1,640,000 | PE |
| Organic Capacity - Current loading - peak week load | 2,278,887 | PE |
| DWF | 4.6 | m^3^/s |
| Average flow | 5.7 | m^3^/s |
| Full flow to Treatment (Design Flow) | 11.1 | m^3^/s |
| Stormwater Flow | 11.5 | m^3^/s |
| Peak flow to Site | 22.6 | m^3^/s |
| Peak Hydraulic Capacity- As Constructed | 959,040 | m^3^/day |
| DWF to the Treatment Plant | 274,076 | m^3^/day |
| Average Hydraulic loading to the Treatment Plant | 458,641 | m^3^/day |
| Average DWF | 492,480 | m^3^/d |
| **Reactor size** | | |
| No. Reactor Basins | 24 |  |
| Volume of one SBR | 13,993 | m³ |
| Total Process Volume | 335,832 | m³ |
| **Carbonaceous Operation** | | |
| Design Sludge Age (total) | 5 | days |
| Design Sludge Age (aerobic) | 4 | days |
| Design MLSS | 2,500 | mg/L |
| **Nitrification** | | |
| Design Sludge Age (total) | 15-25 | days |
| Design Sludge Age (aerobic) | 15 | days |
| Design MLSS | 4,000 | mg/L |
| **Design loads** | | |
| Design BOD Loads | 73,212-98,400 | kg BOD/d |
| Design TKN Loads | 15,600-16,064 | kg TKN/d |
| Design NH3-N Loads | 11,044 | kg NH3.N/d |
| Design TSS Loads | 101,100 | kg TSS/d |

This data was retrieved from the Environmental Protection Agency (EPA) Ireland website, available at <http://www.epa.ie/licences/lic_eDMS/090151b2801ee419.pdf>, accessed on 1 December 2024.

**Table S2** Monthly averages of the influent and effluent from the full-scale aerobic granular sludge unit in Ringsend, Ireland.

| **Parameter** | **Unit** | **Influent** | **Jul** | **Aug** | **Sep** |
| --- | --- | --- | --- | --- | --- |
| BOD | [mg/l] | 224 | 16.25 | 14.25 | 14.5 |
| COD | [mg/l] | 506 | 60 | 54.89 | 79.25 |
| TN | [mg/l as N] | 49.4 | 8.42 | 5.69 | 6.81 |
| Ammonia | [mg/l as N] | 39 | 3.88 | 0.96 | 3.16 |
| Phosphorus (React) | [mg/l as P] | 4.4 | 0.35 | 0.24 | 0.4 |
| TP | [mg/l as P] | 6.7 | 0.89 | 0.67 | 0.88 |

Influent data obtained from 2022 annual report; <https://www.water.ie/sites/default/files/docs/aers/2022/D0034-01_2022_AER.pdf>

**Table S3** Measurements of total suspended solids (TSS), particle size distribution (PSD) and sludge volume index (SVI) for MLSS, influent, effluent, and excess sludge samples collected from Nereda AGS reactor.

| **Sample** | **Size** | **TSS (mg/l)** | **PSD (%)** | **SVI-5 min (ml/g)** | **SVI-30 min (ml/g)** |
| --- | --- | --- | --- | --- | --- |
| MLSS | LG | 825.9 | 21.9 |  |  |
| MLSS | SG | 1990.0 | 52.8 |  |  |
| MLSS | Fl | 951.9 | 25.3 |  |  |
| MLSS | Mix-calc | 3767.8 |  |  |  |
| MLSS | Mix-real | 3860.0 |  | 103.6 | 59.6 |

**Table S4** Metagenomic sequencing processing data/statistics and quality

| **Sample ID** | **Sample type** | **Raw reads** | **Raw Data (bp)** | **Clean reads (%)** | **Q30(%)** | **GC(%)** | **Mapped reads(%)** | **Binned reads(%)** |
| --- | --- | --- | --- | --- | --- | --- | --- | --- |
| G-1 | IN | 69,181,294 | 10,377,194,100 | 98.29 | 93.2 | 46.71 | 46.8 | 36.4 |
| G-2 | FL | 70,198,552 | 10,529,782,800 | 97.82 | 92.77 | 51.23 | 50.8 | 38.4 |
| G-3 | SG | 71,822,878 | 10,773,431,700 | 99.19 | 92.7 | 55.24 | 60.2 | 46.2 |
| G-4 | LG | 72,338,874 | 10,850,831,100 | 99.14 | 93.46 | 56.25 | 51.8 | 38.8 |
| Total/avg |  | 283,541,598 | 42,531,239,700 | 98.61 | 93.03 | 52.36 | 52.4 | 39.95 |

**Table S5** Metatranscriptomics sequencing processing data/statistics and quality.

| **Sample -ID** | **Sample type** | **Raw reads** | **Raw Data (Gp)** | **Clean reads (%)** | **Q30 (%)** | **GC (%)** | **Binned reads (%)** |
| --- | --- | --- | --- | --- | --- | --- | --- |
| T-1 | IN_R1 | 68704788 | 10.31 | 99.6 | 95.32 | 51.9 | 24.20 |
| T-2 | IN_R2 | 71042740 | 10.66 | 99.6 | 95.43 | 50.09 | 23.02 |
| T-3 | IN_R3 | 69234984 | 10.39 | 99.6 | 95.14 | 50.68 | 34.63 |
| T-4 | FL_An_R1 | 75283978 | 11.29 | 99.6 | 93.14 | 53.6 | 31.32 |
| T-5 | FL_An_R2 | 70412248 | 10.56 | 99.6 | 94.38 | 50.58 | 21.91 |
| T-6 | FL_An_R3 | 66957960 | 10.04 | 99.6 | 93.97 | 47.46 | 20.30 |
| T-7 | FL_Ae_R1 | 65874614 | 9.88 | 99.6 | 92.26 | 47.23 | 31.63 |
| T-8 | FL_Ae_R2 | 83005932 | 12.45 | 99.6 | 93.81 | 51.9 | 32.44 |
| T-9 | FL_Ae_R3 | 69749762 | 10.46 | 99.6 | 94.73 | 50.38 | 40.57 |
| T-10 | SG_An_R1 | 75455404 | 11.32 | 99.6 | 92.28 | 54.47 | 35.35 |
| T-11 | SG_An_R2 | 69290294 | 10.39 | 99.6 | 94.73 | 54.83 | 42.61 |
| T-12 | SG_An_R3 | 66950962 | 10.04 | 99.6 | 94.93 | 51.2 | 35.47 |
| T-13 | SG_Ae_R1 | 68888140 | 10.33 | 99.6 | 94.05 | 54.43 | 37.03 |
| T-14 | SG_Ae_R2 | 70115436 | 10.52 | 99.6 | 93.17 | 56.56 | 51.00 |
| T-15 | SG_Ae_R3 | 70524314 | 10.58 | 99.6 | 94.86 | 52.04 | 39.18 |
| T-16 | LG_An_R1 | 70342148 | 10.55 | 99.6 | 93.57 | 51.71 | 28.60 |
| T-17 | LG_An_R2 | 66952572 | 10.04 | 99.6 | 93.42 | 50.36 | 25.65 |
| T-18 | LG_An_R3 | 74905598 | 11.24 | 99.6 | 94.65 | 46.8 | 20.64 |
| T-19 | LG_Ae_R1 | 69023326 | 10.35 | 99.6 | 94.97 | 56.03 | 42.63 |
| T-20 | LG_Ae_R2 | 78250502 | 11.74 | 99.6 | 93.35 | 54.64 | 28.62 |
| T-21 | LG_Ae_R3 | 68777440 | 10.32 | 99.6 | 94.56 | 50.25 | 26.64 |
| Total/avg |  | 1489743142 | 223.46 | 99.60 | 94.13 | 51.77 | 32.07 |

**Table S6** Properties, statistics, and taxonomy of the top 50 high-quality genomes (out of 285 genomes) recovered from the process of assembly and binning.

| **MAG ID** | **Length (Mbp)** | **Completion (%)** | **Redundancy (%)** | **Class** | **Order** | **Family** | **Genus** | **Species** |
| --- | --- | --- | --- | --- | --- | --- | --- | --- |
| MAG_1 | 3.7 | 100 | 0 | Gammaproteobacteria | Xanthomonadales | Xanthomonadaceae | Aquimonas | sp002068275 |
| MAG_2 | 3.0 | 100 | 2.82 | Bacteroidia | Bacteroidales | Prolixibacteraceae | Draconibacterium |  |
| MAG_3 | 1.8 | 100 | 4.23 | Bacteroidia | Cytophagales | Cyclobacteriaceae | ELB16-189 | sp016709845 |
| MAG_4 | 3.4 | 100 | 5.63 | Alphaproteobacteria | Rhizobiales | Aestuariivirgaceae | Aestuariivirga |  |
| MAG_5 | 8.5 | 98.59 | 5.63 | Polyangia | Nannocystales | Nannocystaceae |  |  |
| MAG_6 | 2.8 | 97.18 | 1.41 | Campylobacteria | Campylobacterales | Sulfurospirillaceae | Sulfurospirillum |  |
| MAG_7 | 2.3 | 97.18 | 1.41 | Actinomycetia | Nanopelagicales | UBA10799 | UBA10799 |  |
| MAG_8 | 3.5 | 97.18 | 1.41 | Actinomycetia | Actinomycetales | Dermatophilaceae | Tetrasphaera |  |
| MAG_9 | 4.0 | 97.18 | 2.82 | Acidimicrobiia | Acidimicrobiales | Microtrichaceae | JADJBW01 | sp016703525 |
| MAG_10 | 2.9 | 97.18 | 4.23 | Alphaproteobacteria | Sphingomonadales | Sphingomonadaceae | Sphingorhabdus | lacus |
| MAG_11 | 4.1 | 97.18 | 5.63 | Alphaproteobacteria | Dongiales | Dongiaceae | Dongia |  |
| MAG_12 | 3.9 | 97.18 | 7.04 | Gammaproteobacteria | Burkholderiales | Burkholderiaceae | Rubrivivax |  |
| MAG_13 | 3.7 | 95.77 | 2.82 | Acidimicrobiia | Acidimicrobiales | Microtrichaceae | Ca_Microthrix | parvicella |
| MAG_14 | 4.0 | 95.77 | 2.82 | Anaerolineae | Anaerolineales | EnvOPS12 | OLB14 | sp016715195 |
| MAG_15 | 3.6 | 95.77 | 2.82 | Bacteroidia | Chitinophagales | Saprospiraceae | UBA3362 | sp016710585 |
| MAG_16 | 3.5 | 95.77 | 4.23 | Bacteroidia | Chitinophagales | JADIYW01 | JADJSS01 | sp016721245 |
| MAG_17 | 3.7 | 95.77 | 5.63 | Bacteroidia | Flavobacteriales | PHOS-HE28 | PHOS-HE28 | sp016721825 |
| MAG_18 | 3.8 | 95.77 | 7.04 | Gammaproteobacteria | Burkholderiales | Rhodocyclaceae | Ca_Accumulibacter | propinquus |
| MAG_19 | 4.3 | 95.77 | 8.45 | Alphaproteobacteria | Sphingomonadales | Sphingomonadaceae | Chakrabartia | sp016714795 |
| MAG_20 | 7.3 | 95.77 | 9.86 | Gammaproteobacteria | Burkholderiales | Rhodocyclaceae | Ca_Accumulibacter |  |
| MAG_21 | 2.6 | 94.37 | 0 | Clostridia | Lachnospirales | Lachnospiraceae | CAG-791 |  |
| MAG_22 | 3.3 | 94.37 | 1.41 | Actinomycetia | Actinomycetales | Dermatophilaceae | Tetrasphaera | Ca. Phosphoribacter |
| MAG_23 | 3.4 | 94.37 | 1.41 | Bacteroidia | AKYH767-A | OLB10 | CAINEC01 | sp017995075 |
| MAG_24 | 4.1 | 94.37 | 2.82 | Bacteroidia | AKYH767 | B-17BO | UBA2475 |  |
| MAG_25 | 2.5 | 94.37 | 2.82 | Bacteroidia | Chitinophagales | Chitinophagaceae | Terrimonas | sp016706055 |
| MAG_26 | 3.9 | 94.37 | 2.82 | Gammaproteobacteria | Pseudomonadales | UBA5518 | UBA5518 |  |
| MAG_27 | 1.9 | 94.37 | 2.82 | Gammaproteobacteria | Burkholderiales | Burkholderiaceae | CAISIP01 | sp018063505 |
| MAG_28 | 3.5 | 94.37 | 5.63 | Gammaproteobacteria | Burkholderiales | Rhodocyclaceae | Methyloversatilis |  |
| MAG_29 | 7.9 | 94.37 | 5.63 | Myxococcia | Myxococcales | Myxococcaceae | JAEUJQ01 |  |
| MAG_30 | 3.2 | 94.37 | 5.63 | Gammaproteobacteria | Competibacterales | Competibacteraceae | Ca_Contendobacter | odensis_A |
| MAG_31 | 2.9 | 92.96 | 0 | Gammaproteobacteria | Xanthomonadales | Ahniellaceae | JADKHK01 | sp016721845 |
| MAG_32 | 2.4 | 92.96 | 1.41 | Bacteroidia | Chitinophagales | JADIYW01 | JADJSS01 |  |
| MAG_33 | 2.6 | 92.96 | 1.41 | Bacteroidia | NS11-12g | UKL13-3 | UBA6183 | sp018058165 |
| MAG_34 | 3.6 | 92.96 | 1.41 | UBA1135 | UBA2386 | UBA2386 |  |  |
| MAG_35 | 5.8 | 92.96 | 2.82 | Acidimicrobiia | Acidimicrobiales | Microtrichaceae | AWTP1-35 |  |
| MAG_36 | 4.6 | 92.96 | 2.82 | Bacteroidia | Chitinophagales | JADIYW01 |  |  |
| MAG_37 | 6.4 | 92.96 | 4.23 | Vampirovibrionia | Obscuribacterales | Obscuribacteraceae | Ga0077546 |  |
| MAG_38 | 2.3 | 92.96 | 4.23 | Bacteroidia | AKYH767 | B-17BO | UBA2475 |  |
| MAG_39 | 4.0 | 92.96 | 4.23 | Bacteroidia | AKYH767 | B-17BO | UBA4416 |  |
| MAG_40 | 3.6 | 92.96 | 4.23 | Gammaproteobacteria | Burkholderiales | Rhodocyclaceae | Azonexus | sp017983615 |
| MAG_41 | 3.0 | 92.96 | 5.63 | Gammaproteobacteria | Burkholderiales | Rhodocyclaceae | Propionivibrio | sp016705685 |
| MAG_42 | 6.6 | 92.96 | 7.04 | Polyangia | Polyangiales | Polyangiaceae |  |  |
| MAG_43 | 5.8 | 92.96 | 9.86 | Polyangia | Polyangiales | Ga0077539 | JAEUKH01 |  |
| MAG_44 | 3.3 | 92.96 | 9.86 | Bacteroidia | Cytophagales | UBA9547 | UBA9547 |  |
| MAG_45 | 4.2 | 92.96 | 9.86 | Gammaproteobacteria | Burkholderiales | Rhodocyclaceae | Ca_Accumulibacter |  |
| MAG_46 | 2.6 | 91.55 | 1.41 | Bacteroidia | Flavobacteriales | Flavobacteriaceae | Flavobacterium |  |
| MAG_47 | 3.8 | 91.55 | 2.82 | Bacteroidia | Flavobacteriales | PHOS-HE28 | PHOS-HE28 | sp016710685 |
| MAG_48 | 3.3 | 91.55 | 2.82 | Bacteroidia | Flavobacteriales | PHOS-HE28 | PHOS-HE28 | sp016719225 |
| MAG_49 | 3.8 | 91.55 | 4.23 | Holophagae | Holophagales | Holophagaceae | JACQZU01 |  |
| MAG_50 | 3.7 | 91.55 | 4.23 | Bacteroidia | NS11-12g | UBA9320 | JABWCE01 |  |


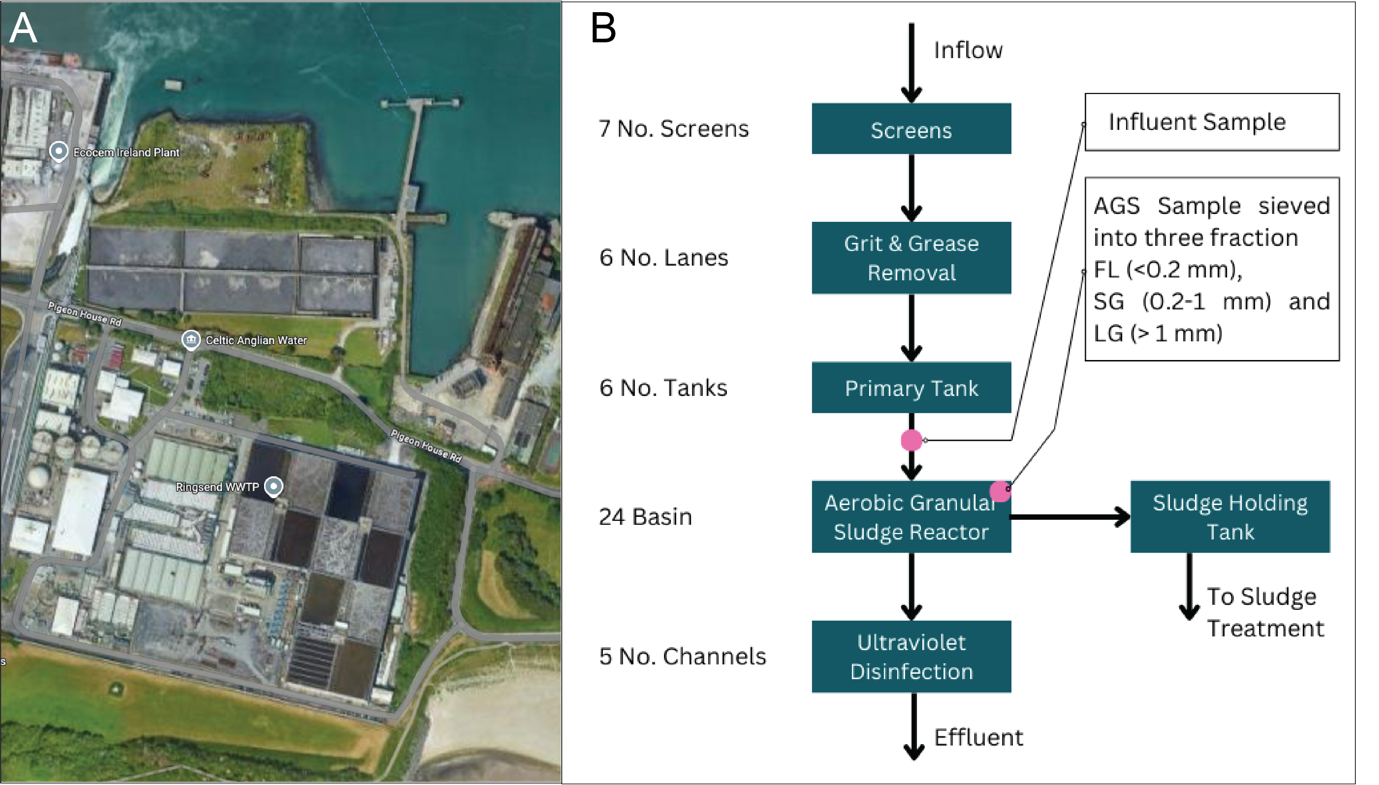


**Fig. S1** (A) The satellite map of Ringsend wastewater treatment plant in Ireland. The image was downloaded from Google Maps on 28 Dec, 2024. The satellite map shows the 24 tanks constructed on two levels (12 tanks per floor). (B) The schematic overview of the treatment flow of Ringsend wastewater treatment plant. This data was retrieved from the Environmental Protection Agency (EPA) Ireland website, available at <http://www.epa.ie/licences/lic_eDMS/090151b2801ee419.pdf>, accessed on 1 December 2024. Pink dots represent the sampling points of the study.

| 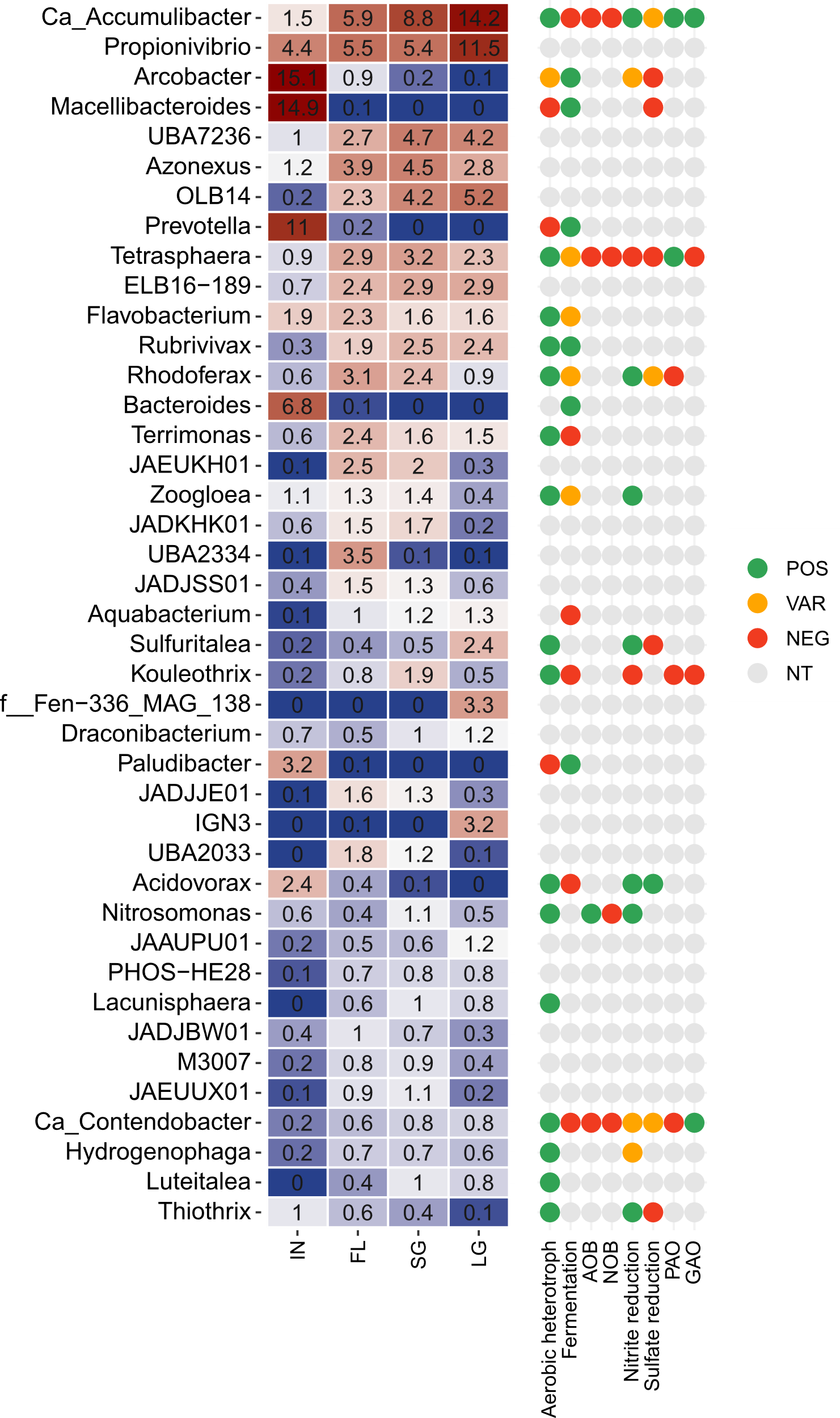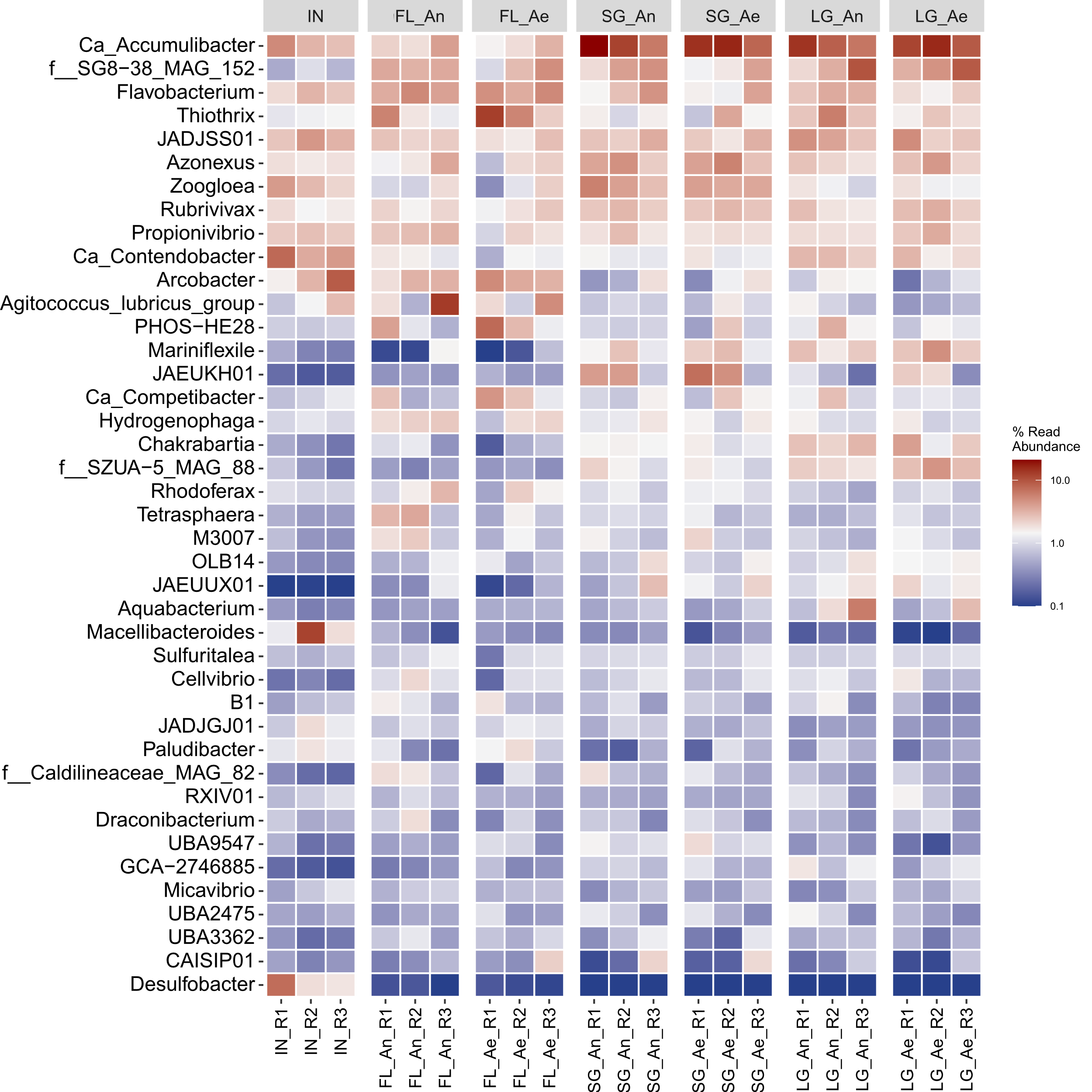  **A.**  **B.** |
| --- |

**Fig. S2** Relative abundances (%) of the top 40 genera in influent wastewater (IN), flocs (FL), small granules (SG), and large granules (LG) samples based on: A) metagenomics data (n=1); B) metatranscriptomics data (n=3). Metatranscriptomics heatmap for FL, SG, and LG includes both anaerobic (An) and aerobic conditions (Ae).

| 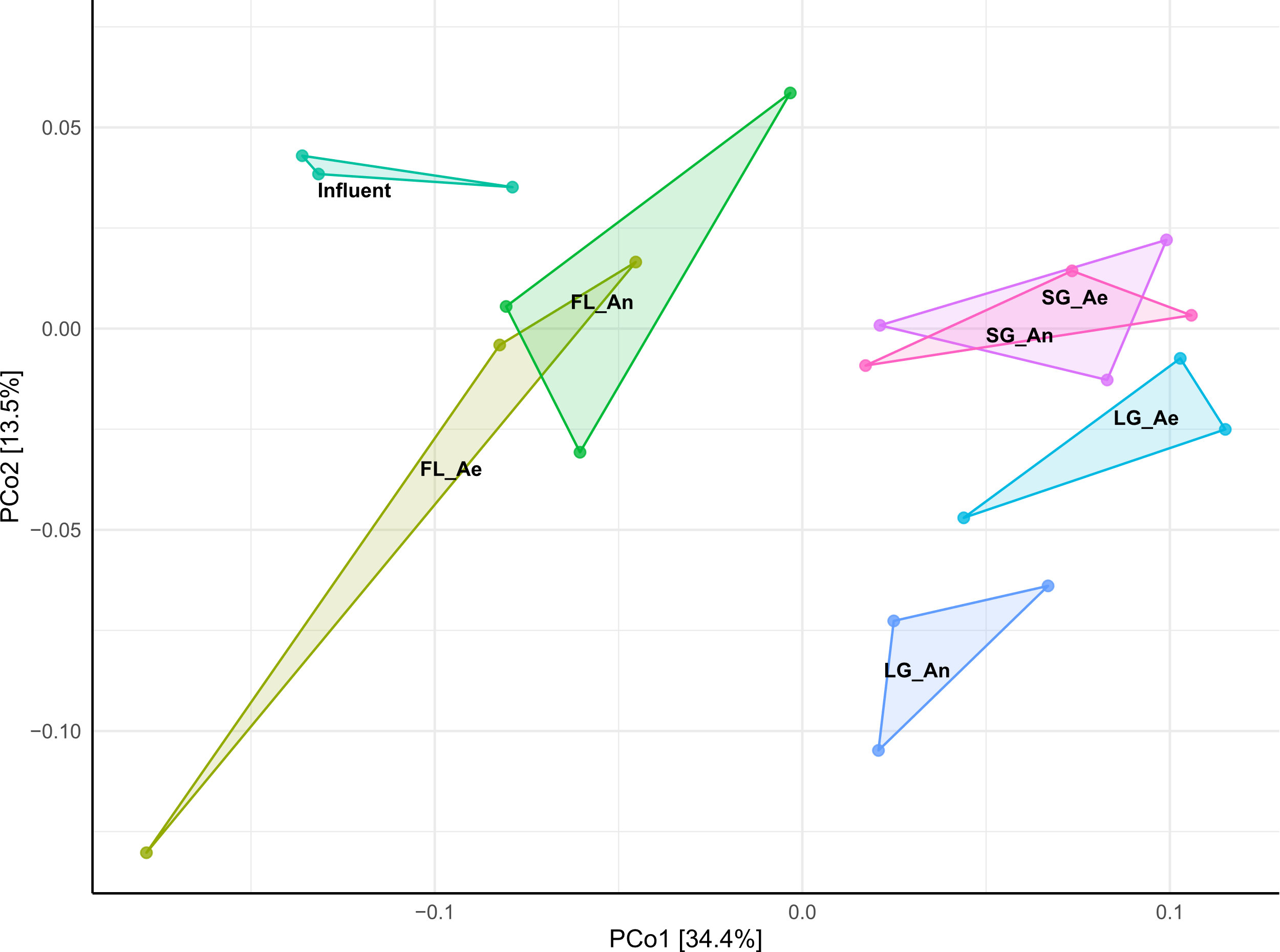  **A.** | 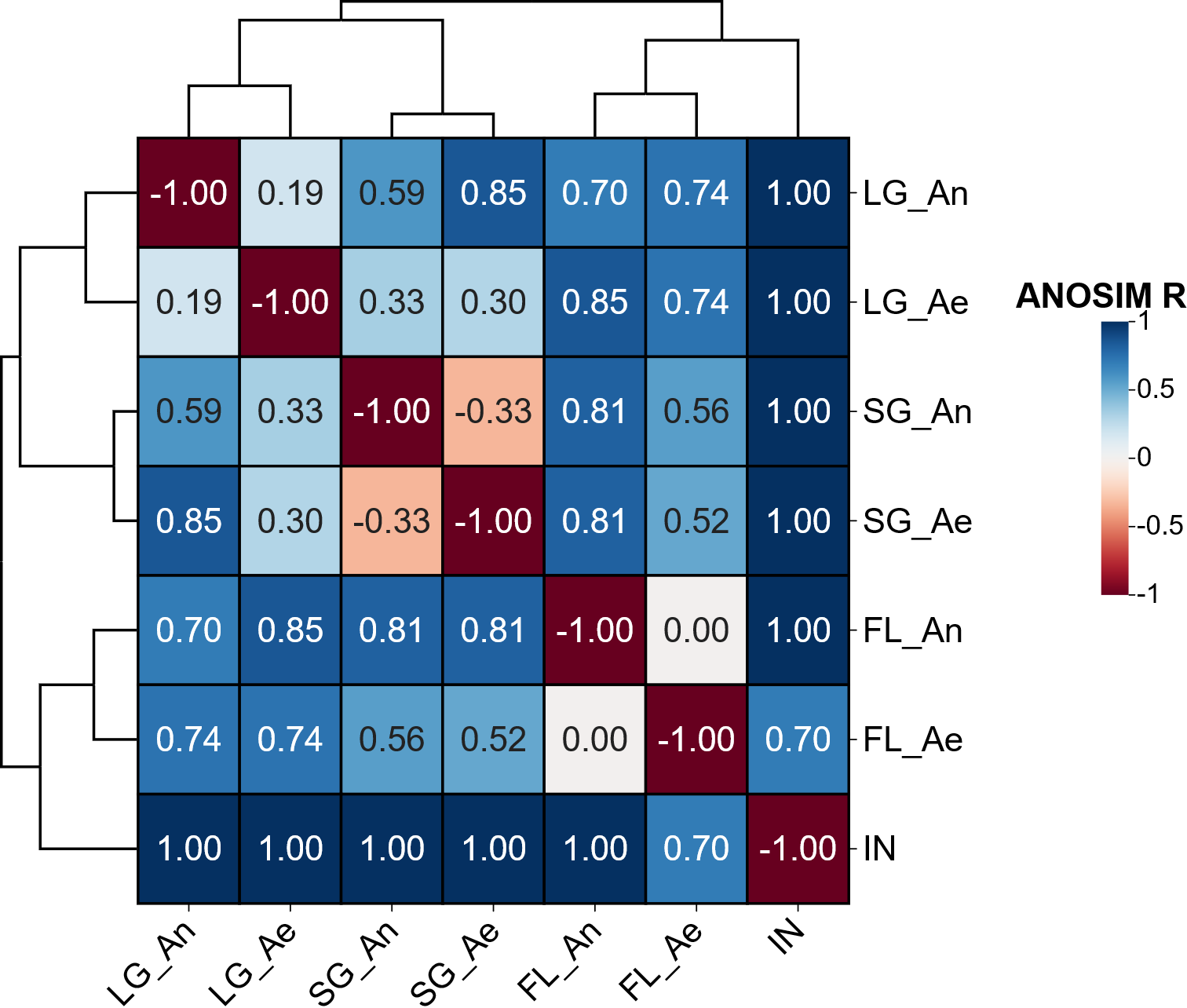  **B.** |
| --- | --- |

**Fig. S3** Metatranscriptomics based community composition (Beta diversity): A) Principal Coordinates Analysis (PCoA) showing similarity between influent wastewater (IN), flocs (FL), small granules (SG), and large granules (LG) based on distance metric (Bray-Curtis); B) analysis of similarity (ANOSIM) (falls between -1 and 1). A positive R value means that inter-group variation is considered significant, while a negative R-value suggests that inner-group variation is larger than inter-group variation, therefore, no significant differences.

|  | 1. Genomic Level | 1. Genes level |
| --- | --- | --- |
| Flocs (FL) | 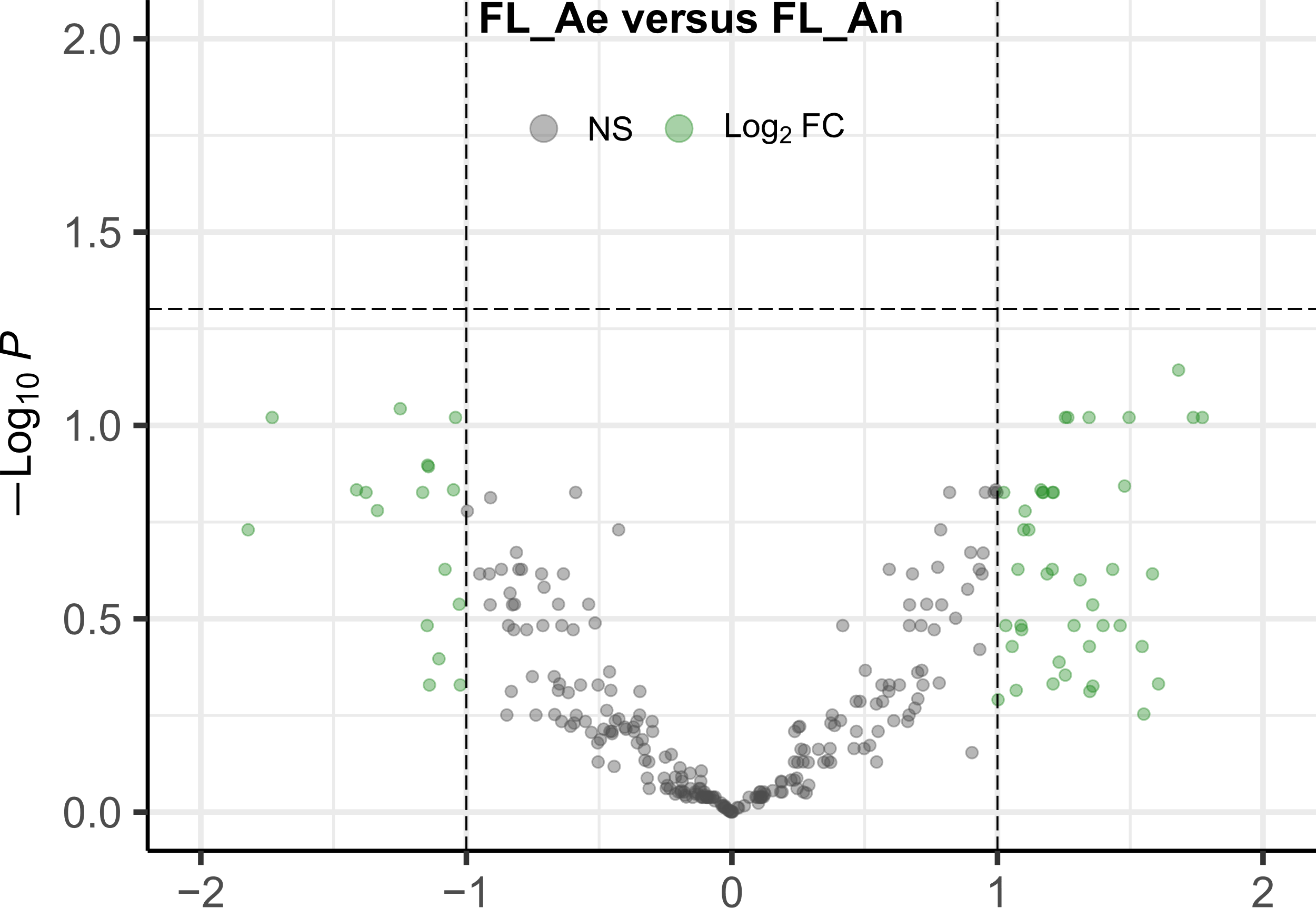 | 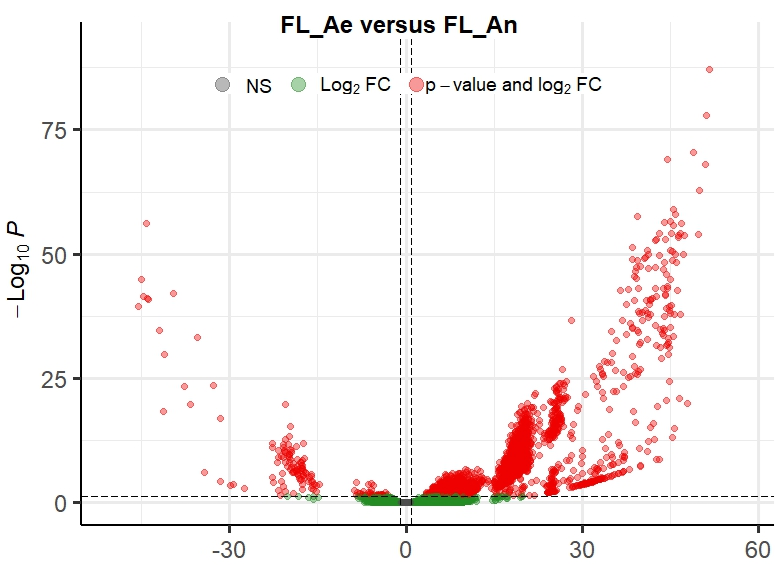 |
| Small granules (SG) | 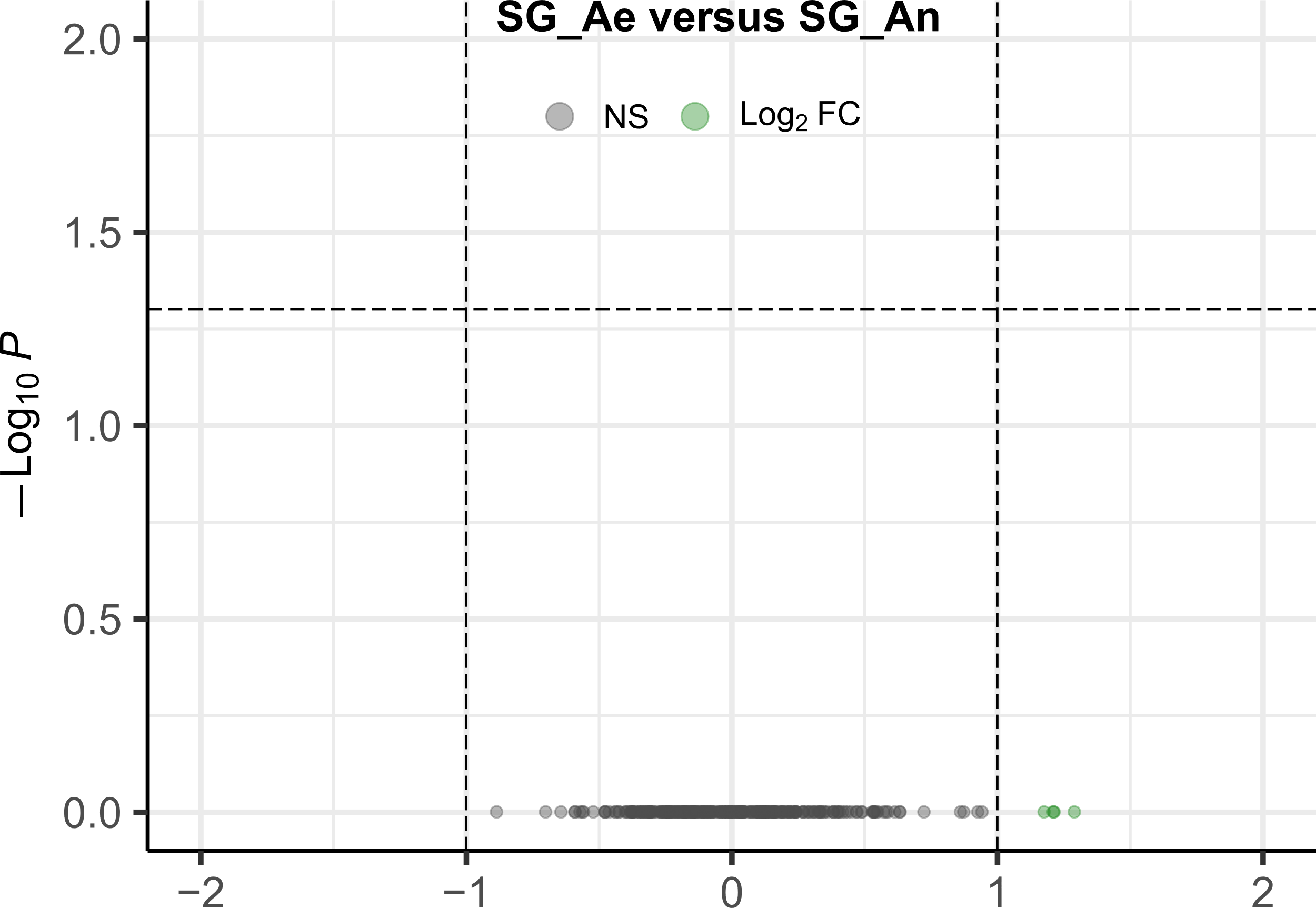 | 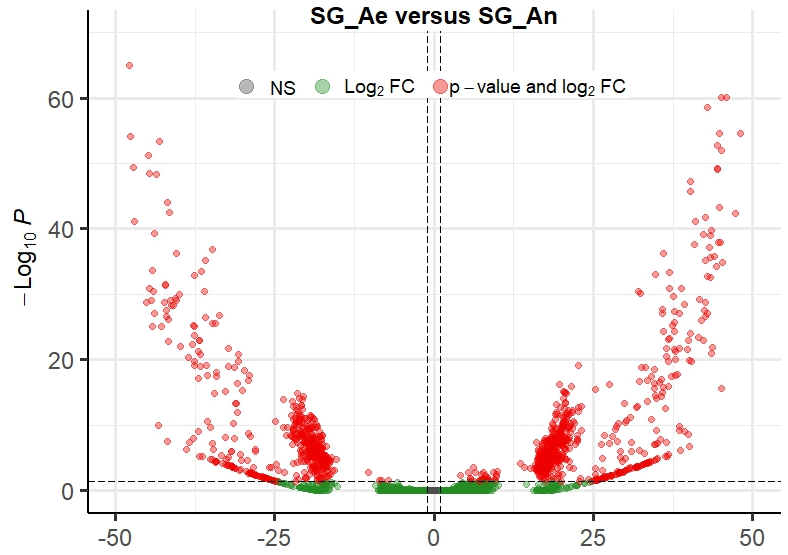 |
| Large granules (LG) | 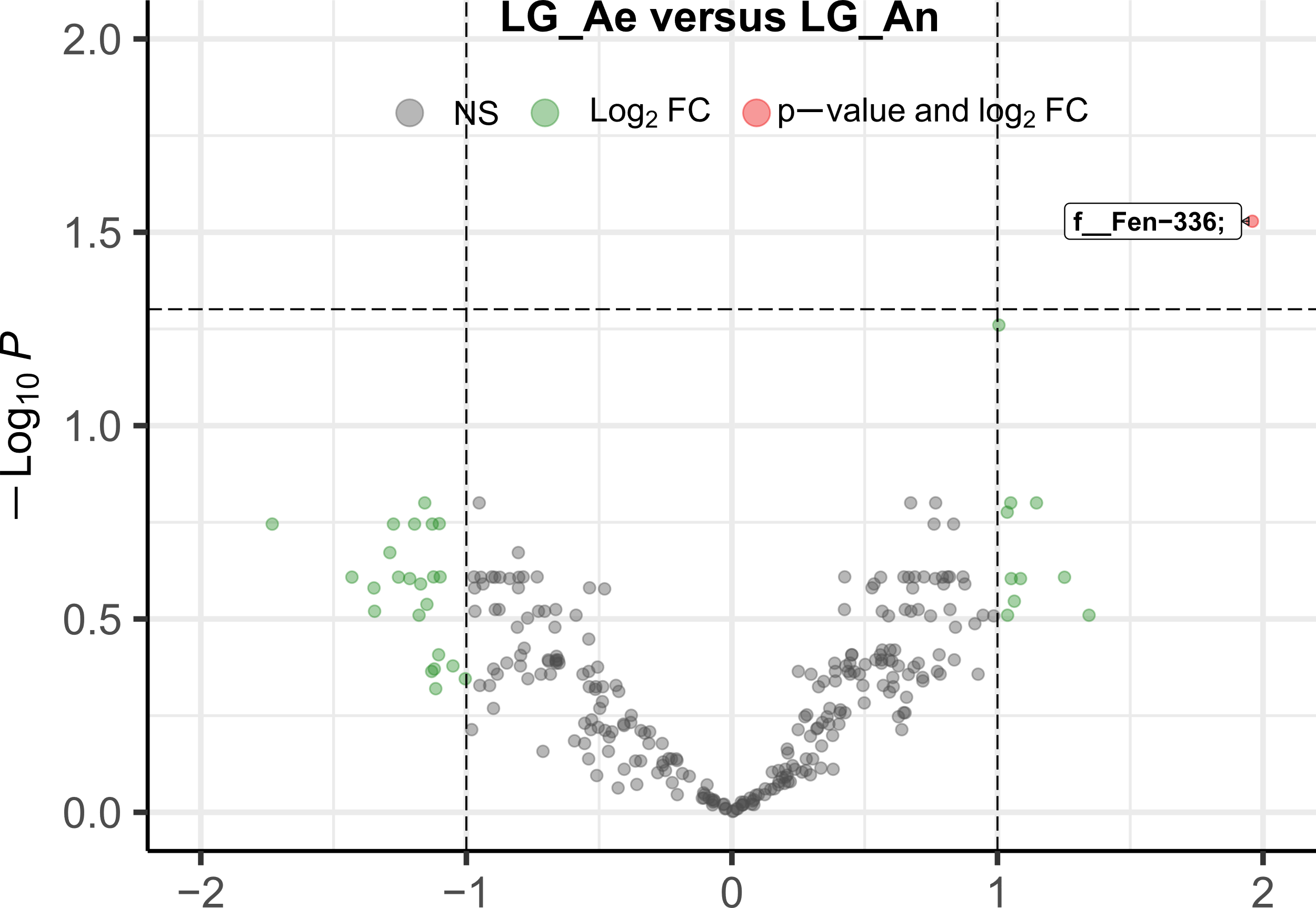 | 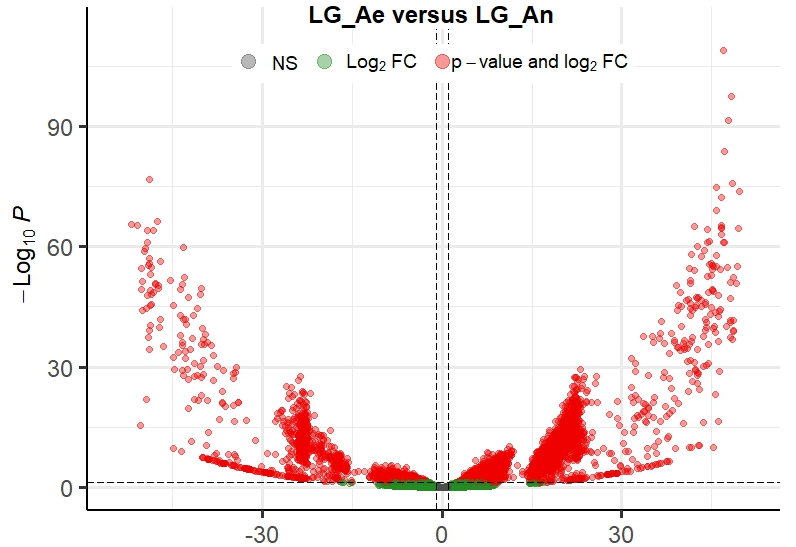 |
| Global | 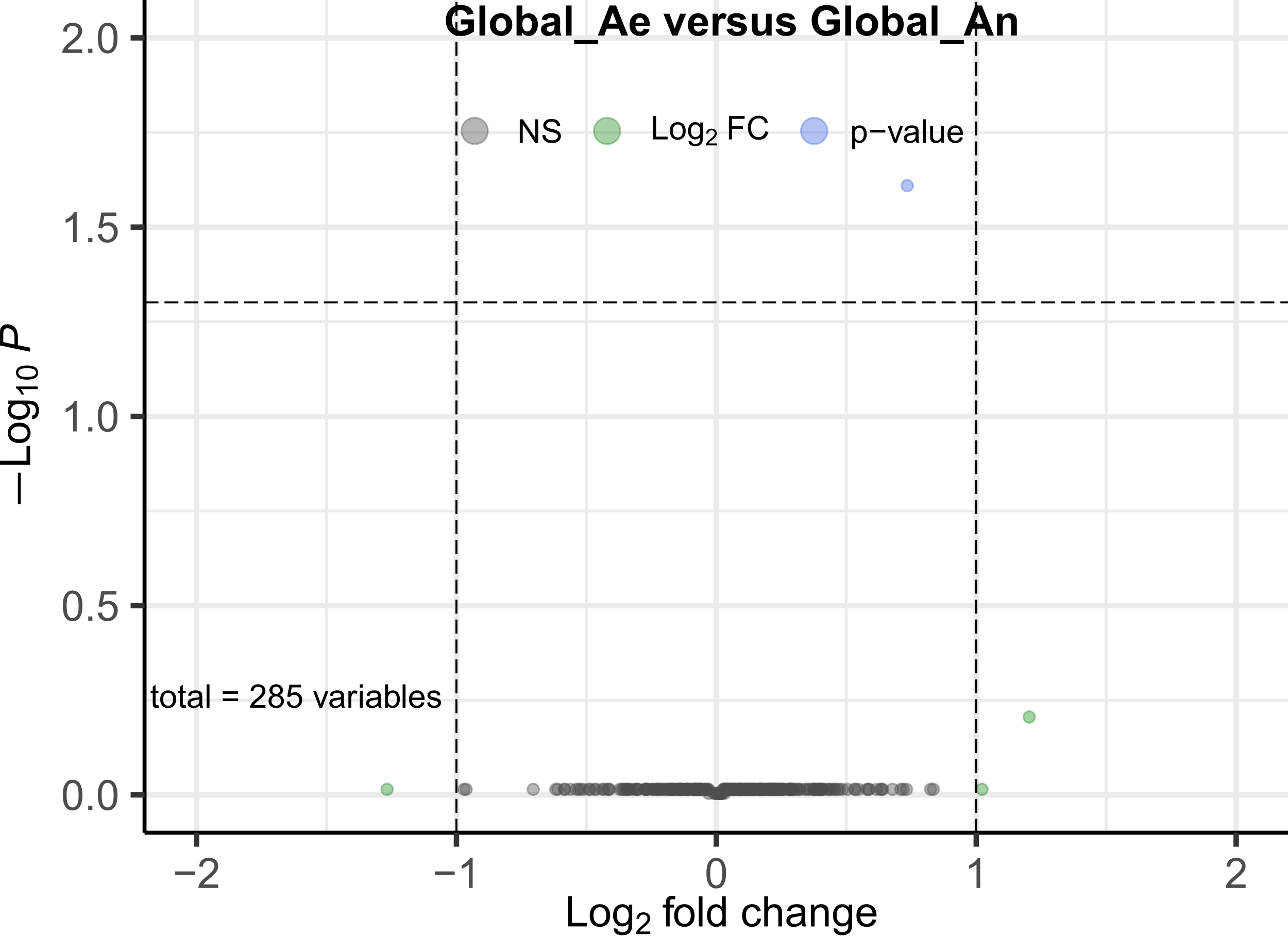 | 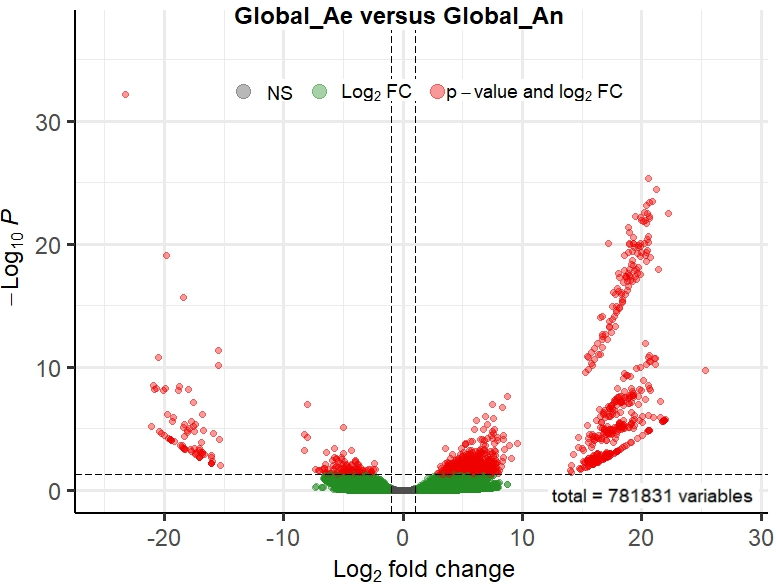 |

**Fig. S4** EnhancedVolcano plots showing differential expression analysis between aerobic (Ae) and anaerobic (An) conditions for varied-sized microbial aggregates (flocs (FL), small granules (SG), large granule (LG), and global) using DESeq2. A) based on genomic level (285 genomes); B) based on genes level (781831 genes). Species were coloured based on the level of significance and degrees of variation; either as absolute log_2_ fold change |log_2_ FC| ≥ 1 or *P*-value < 5% or meeting both |log_2_ FC| ≥ 1 and P-value < 5%, or meeting none of the previous condition as non-significant (NS). Genomes and genes with negative log_2_ FC indicate they were downregulated (less active) aerobically, while positive log_2_ FC values indicate they were upregulated (more active) aerobically. Y-axis is on – log_10_ P value; so, P value of 5% (0.05) equates to 1.3.

| **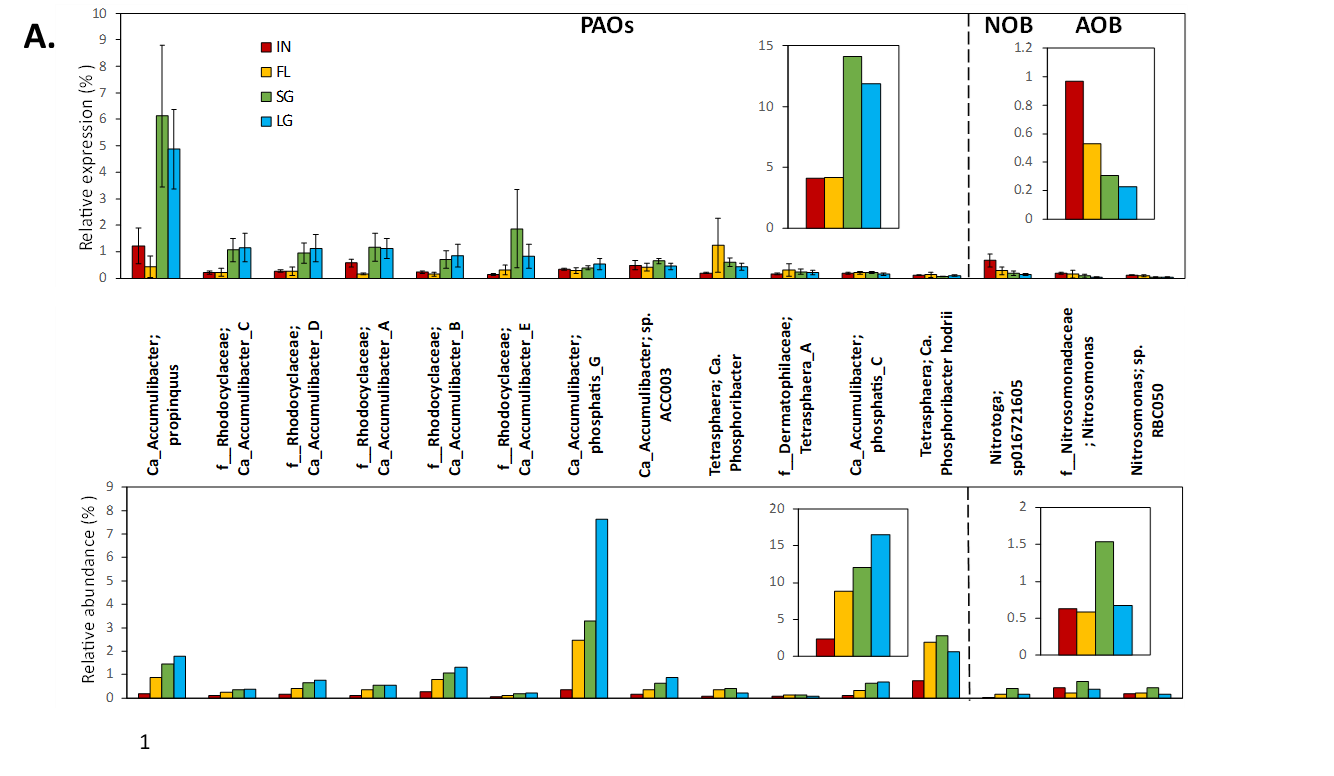**  **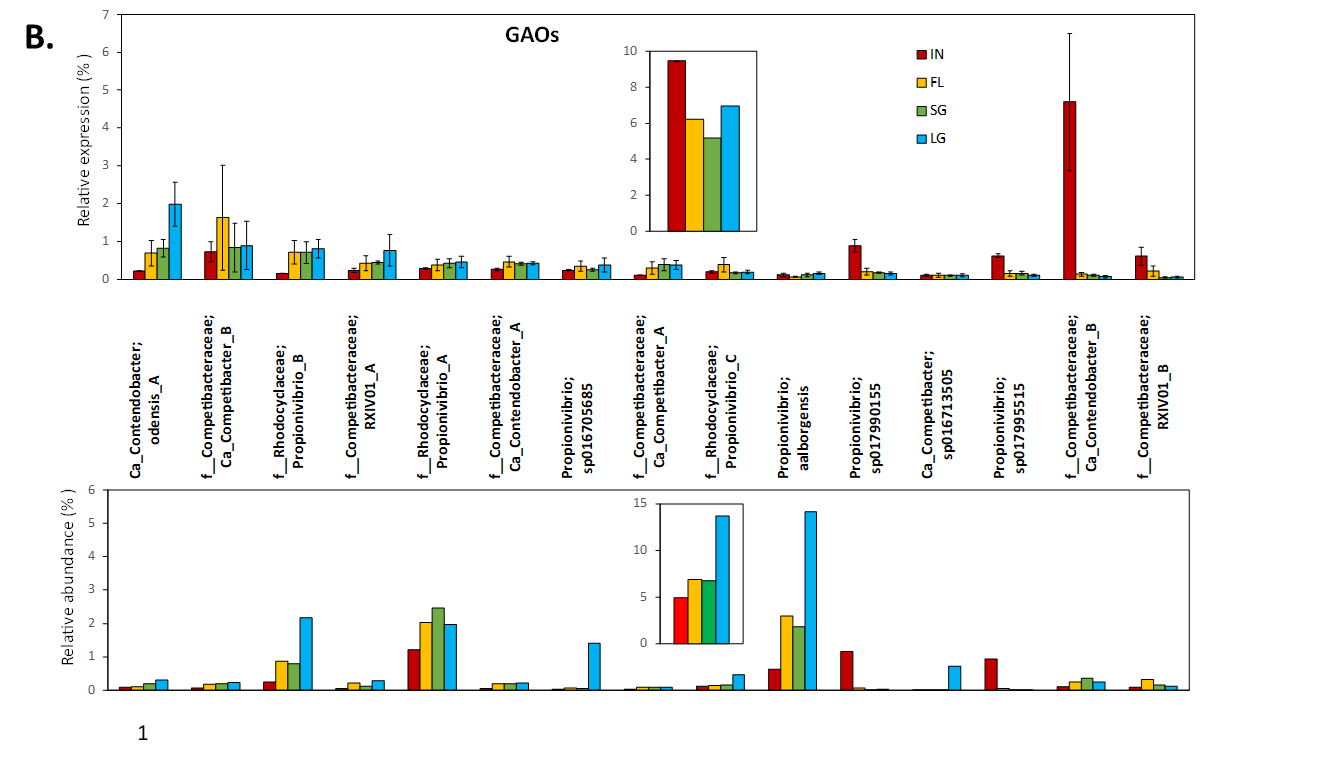** |
| --- |

**Fig. S5** Transcriptomics relative expression (mean ± SD, n=3) and MAGs relative read abundance for species of the important functional groups in influent, flocs (FL), small granules (SG) and large granules (LG). **A)** PAOs and nitrifiers (AOB and NOB); and **B)** GAOs. The species were ordered from the highest to lowest transcriptomics relative expression in LG samples. Anaerobic and aerobic values of FL, SG and LG were averaged as there were no significant differences in transcription activities at the genome-level between the two conditions **(Fig. S3)**. The box inside each graph shows the cumulative relative expressions or MAG relative abundances of species per each functional group.
